# Single-cell RNA-Seq analysis of diabetic wound macrophages in STZ-induced mice

**DOI:** 10.1007/s12079-022-00707-w

**Published:** 2022-11-29

**Authors:** Jiaxu Ma, Ru Song, Chunyan Liu, Guoqi Cao, Guang Zhang, Zhenjie Wu, Huayu Zhang, Rui Sun, Aoyu Chen, Yibing Wang, Siyuan Yin

**Affiliations:** 1 Department of Plastic Surgery, Cheeloo College of Medicine, Shandong Provincial Qianfoshan Hospital, Shandong University, 250012 Jinan, Shandong P. R. China; 2 Department of Plastic Surgery, Shandong Provincial Qianfoshan Hospital, The First Affiliated Hospital of Shandong First Medical University, 250014 Jinan, Shandong P. R. China; 3 Jinan Clinical Research Center for Tissue Engineering Skin Regeneration and Wound Repair, The First Affiliated Hospital of Shandong First Medical University & Shandong Provincial Qianfoshan Hospital, 250014 Jinan, P. R. China

**Keywords:** Macrophage, Single-cell RNA sequencing, Chronic diabetic wound.

## Abstract

The crucial role of macrophages in the healing of chronic diabetic wounds is widely known, but previous in vitro classification and marker genes of macrophages may not be fully applicable to cells in the microenvironment of chronic wounds. The heterogeneity of macrophages was studied and classified at the single-cell level in a chronic wound model. We performed single-cell sequencing of CD45 + immune cells within the wound edge and obtained 17 clusters of cells, including 4 clusters of macrophages. One of these clusters is a previously undescribed population of macrophages possessing osteoclast gene expression, for which analysis of differential genes revealed possible functions. We also analysed the differences in gene expression between groups of macrophages in the control and diabetic wound groups at different sampling times. We described the differentiation profile of mononuclear macrophages, which has provided an important reference for the study of immune-related mechanisms in diabetic chronic wounds.

**Graphical Abstract:**

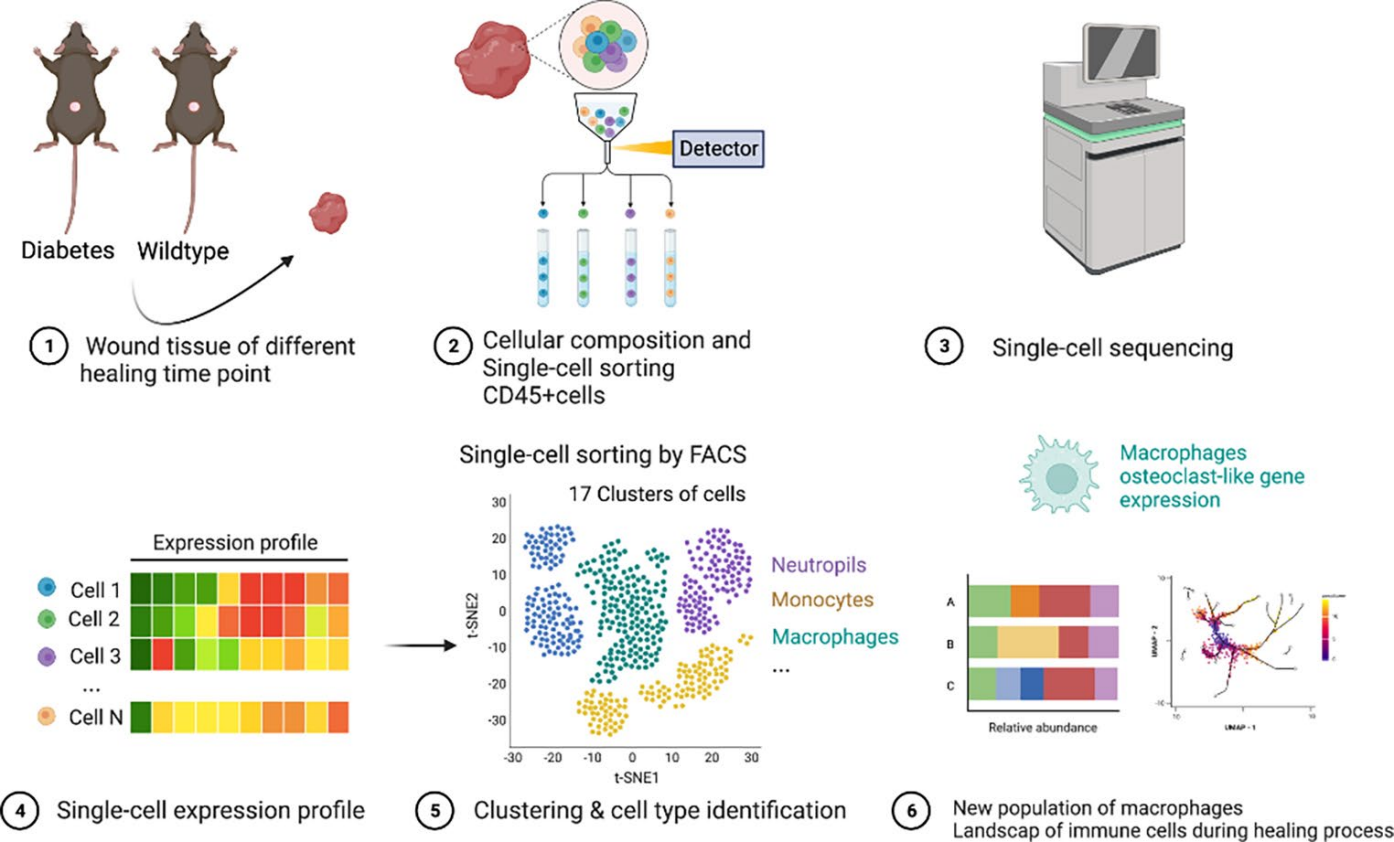

**Supplementary information:**

The online version contains supplementary material available at 10.1007/s12079-022-00707-w.

## Background

The clinical treatments for chronic wound remain ineffective, and the dramatic increase in healthcare costs has created a heavy financial strain, with total US Medicare treatment costs for wound-related care ranging from $28.1 to $96.8 billion in 2018 statistics, in which diabetic ulcers and surgical wound were the most costly (Nussbaum et al. 2018). From the 2017–2020 US reported statistics, the 5-year survival rate for diabetic foot was 30.5%, which was similar to the 5-year survival rate for cancer combined of 31%, while in terms of care, diabetes care was more expensive, and a significant amount of funding was consumed for lower extremity care (Armstrong et al. 2020). The ageing population has a high prevalence of chronic wound, and ageing is becoming an important worldwide healthcare and demographic issue (OECD 2014). Projections indicate that in 2050, there will be more older people aged 60 years or older than adolescents aged 10–24 years (2.1 billion versus 2 billion) (Rudnicka et al. 2020). The global market for advanced wound care is expected to reach US$18.7 billion by 2027, growing at a compound annual growth rate (CAGR) of 6.6% during the analysis period 2020–2027 (Sen 2021). Diabetic patients and obese individuals are at high risk of chronic wounds. Chronic inflammation is also an important cause of chronic wound in diabetes due to the long-term effects of high sugar and free fatty acids, which lead to a chronic inflammatory state in a variety of tissues. Chronic inflammation in diabetes can be manifested by the recruitment of immune cells such as macrophages and neutrophils to tissues and the release of proinflammatory cytokines (Pahwa et al. 2021). The current classification of macrophages as an important part of immune cells has revealed some differences in vitro and in vivo, and the previous markers for typing these cells are not well suited for the current study (Orecchioni et al. 2019). Single-cell sequencing, a new technology that has emerged in recent years (Saliba et al. 2014), provides a way to classify and functionally study cell populations in specific microenvironments independent of prior experience (Hedlund and Deng 2018; Stuart and Satija, 2019). Using the BD Rhapsody single-cell platform (Fan et al. 2015; Mair et al. 2020), we performed unbiased analysis of CD45 + immune cells from the skin of STZ-induced C57BL/6J mice and wild-type mice in a whole skin wound model to discover the distribution of the different populations of immune cells, as well as the genetic profile of wound-associated macrophages and analysed their temporal genetic differences.

## Materials and methods

### Animals

In this study, male C57BL/6J mice weighing 20–25 g were used. All experimental protocols were approved by the ethics committee of Shandong Qianfoshan Hospital, Shandong University, and all experiments were performed according to the approved protocols. Mice were housed under controlled temperature (26 ± 1.5 °C) and humidity (60% ± 5) with a 12-h light/dark schedule.

### Development of the diabetic mouse model

Streptozotocin (120 mg/kg, Sigma-Aldrich, USA, S0130) was administered to 4-week-old C57BL6J mice after 12 h of fasting. Blood glucose was measured after 7 days of stabilization, and mice with glucose levels ≥ 16.5 mmol/L (300 mg/dL) were considered diabetic and were used for the operation.

### Wound sampling

Diabetic mice (n = 20) and control mice (n = 20) were anesthetized with isoflurane inhalation, and then the hair on the back was removed to create a full skin wound 5 mm in diameter, which was observed daily. The randomly selected mice (n = 5) were separately sacrificed on day 1, day 3, day 5 and day 7, and wound tissue was obtained. The wounds obtained from each group of randomly selected mice (n = 5) were mixed and used for the subsequent preparation of single-cell suspensions.

### Preparation of single-cell suspensions

The digestion solution was composed of collagenase II (3.5 mg/mL, Solarbio, China), DNase I (0.02 mg/mL, Solarbio, China), hyaluronidase (30 U/mL, Solarbio, China), 5% FBS-1640 (Gibco, USA), and DPBS (Gibco, USA). Samples were washed, the digestion solution was added to the tissue, and the tissue was cut and incubated at 37 °C for 1 h. The digestion solution was passed through a 40-µm cell strainer and then centrifuged at 500 x g for 5 min. The reaction was terminated by adding erythrocyte lysate for 5 min and centrifuging twice at 300 x g for 5 min.

### Flow sorting

A portion of the cell suspension was centrifuged at 300 x g for 5 min. 100 µL of wash solution was used to resuspend the cell precipitate, 2 µL of APC anti-mouse CD45 antibody (BioLegend, USA) was added for every 1× 10 ^6 ^cells according to the total number of cells, and the mixture was incubated at 4 °C and protected from light for a total incubation time of 30 min. At 15 min of incubation time, 1 µL of calcein AM (BD, USA) was added for every 100 µL of cell suspension. After incubation, the cells were washed twice by centrifugation at 300 x g for 5 min; the supernatant was discarded, and the cells were resuspended and mixed with the appropriate amount of washing solution. A small amount of the suspension was stained with AO/PI, and the number of cells sorted, the cell viability and the clumping rate were recorded; the cells were centrifuged at 300 x g for 5 min and resuspended in sample buffer for BD quality control.

### Single-cell RNA sequencing

The transcriptomic information of sorted CD45 + cells was captured by BD Rhapsody system. Single-cell was randomly distributed across > 200,000 microwells through a limited dilution approach. Beads with oligonucleotide barcodes were added to saturation so that a bead was paired with a cell in a microwell. The cells were lysed in the microwell to hybridize mRNA molecules to barcoded capture oligos on the beads. Beads were collected into a single tube for reverse transcription and ExoI digestion. Upon cDNA synthesis, each cDNA molecule was tagged on the 5′ end (that is, the 3′ end of a mRNA transcript) with a unique molecular identifier (UMI) and cell barcode indicating its cell of origin. Whole transcriptome libraries were prepared using the BD Rhapsody single-cell whole-transcriptome amplification (WTA) workflow including random priming and extension (RPE), RPE amplification PCR and WTA index PCR. The libraries were quantified using a High Sensitivity DNA chip (Agilent) on a Bioanalyzer 2200 and the Qubit High Sensitivity DNA assay (Thermo Fisher Scientific). Sequencing was performed by illumina sequencer (Illumina, San Diego, CA) on a 150 bp paired-end run.

### Single-cell RNA statistical analysis

scRNA-seq data analysis was performed by NovelBio Co.,Ltd. with NovelBrain Cloud Analysis Platform. We applied fastp with default parameter filtering the adaptor sequence and removed the low quality reads to achieve the clean data. UMI-tools was applied for Single Cell Transcriptome Analysis to identify the cell barcode whitelist. The UMI-based clean data was mapped to mouse genome (Ensemble version 92) utilizing STAR mapping with customized parameter from UMI-tools standard pipeline to obtain the UMIs counts of each sample. Cells contained over 200 expressed genes and mitochondria UMI rate below 20% passed the cell quality filtering and mitochondria genes were removed in the expression table. Seurat package (version: 2.3.4, https://satijalab.org/seurat/) was used for cell normalization and regression based on the expression table according to the UMI counts of each sample and percent of mitochondria rate to obtain the scaled data. PCA was constructed based on the scaled data with top 2000 high variable genes and top 10 principals were used for tSNE construction and UMAP construction.

Utilizing graph-based cluster method (resolution = 0.8), we acquired the unsupervised cell cluster result based the PCA top 10 principal and we calculated the marker genes by FindAllMarkers function with wilcox rank sum test algorithm under following criteria:1. lnFC > 0.25; 2. pvalue < 0.05; 3. min.pct > 0.1. In order to identify the cell type detailed, the clusters of same cell type were selected for re-tSNE analysis, graph-based clustering and marker analysis.

### Cell-cycle discrimination analyses

We used cell cycle-related genes, including a previously defined core set of 43 G1/S and 54 G2/M genes (Tirosh et al. 2016). For each cell, a cell cycle phase (G1, S, G2/M) was assigned based on its expression of G1/S or G2/M phase genes using the scoring strategy described in CellCycleScoring function in Seurat. Cells in each cell cycle state were also quantified using the which.cells function in Seurat.

### Pseudo-Time Analysis

We applied the Single-Cell Trajectories analysis utilizing Monocle2 (http://cole-trapnell-lab.githu.b.io/monocle-release) using DDR-Tree and default parameter. Before Monocle analysis, we select marker genes of the Seurat clustering result and raw expression counts of the cell passed filtering. Based on the pseudo-time analysis, branch expression analysis modeling (BEAM Analysis) was applied for branch fate determined gene analysis.

### Cell Communication Analysis

To enable a systematic analysis of cell-cell communication molecules, we applied cell communication analysis based on the CellPhoneDB, a public repository of ligands, receptors and their interactions. Membrane, secreted and peripheral proteins of the cluster of different time point was annotated. Significant mean and Cell Communication significance (p-value < 0.05) was calculated based on the interaction and the normalized cell matrix achieved by Seurat Normalization.

### QuSAGE Analysis (Gene Enrichment Analysis)

To characterize the relative activation of a given gene set such as pathway activation, we performed QuSAGE (2.16.1) analysis.

## Results

### F1 scRNA-seq based identification of STZ-induced diabetic mouse wounds immune cell populations

**Fig. 1:**
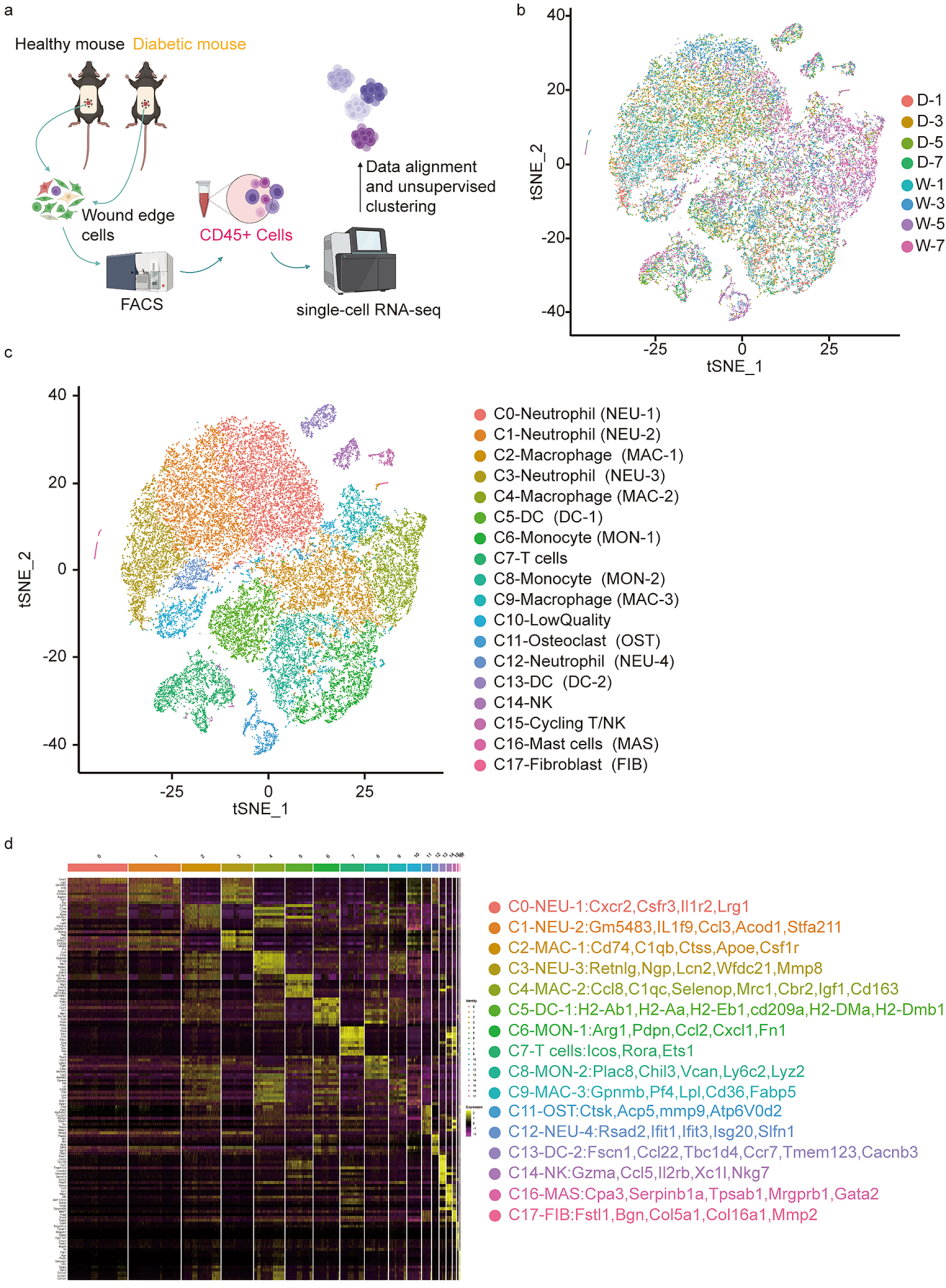
scRNA-seq based identification of STZ-induced diabetic mouse wounds immune cell populations. **(a)** Experimental design. Single cell were collected from day1,day3,day5,day7,along wound healing **(b)** A t-distributed stochastic neighbour embedding (t-SNE) visualization of all cells displayed with different colours for samples **(c)** t-SNE visualization of 9240 single cells, colour-coded by assigned cell type **(d)** Heat map of all clusters top 20 upregulated marker gene. Shades of colour indicate high or low gene expression, with yellow being high expression and dark red being low expression

We performed scRNA-seq on CD45 + cells gathered from wound tissue obtained from wild-type and STZ-induced diabetic C57BL/6J mice (Fig. 1a). Four time points were selected for sampling (1, 3, 5, and 7 days). The single-cell data of the obtained samples were normalized by excluding low-quality cells to eliminate batch effects, and data from a total of 9240 cells were obtained. Principal component analysis (PCA) was performed, and the results were plotted with t-stochastic neighbour embedding (t-SNE) downscaled to show the distribution of cells from different sample sources in the overall data (Fig. 1b), along with the gene expression level of all single cells and the number of their UMI expressed (supplementary1).

QC cell data were unbiased using the Seraut package, and gene expression data from cells extracted from both conditions were aligned and projected in a 2D space through t-SNE to allow identification of overlapping and diabetic wound-associated immune cell populations. A total of 17 cell clusters were obtained, except for low-quality cells, which have a high preponderance of mitochondrial genes (Fig. 1c). We mapped the heat map of major marker genes in all populations (Fig. 1d). The cell populations obtained were 4 clusters of neutrophils (cluster 0, cluster 1, cluster 3 and cluster 12, with marker genes Ptprc, S100a8, s100a9, Csf3r, Cxcr2, and Lrg1); 2 clusters of monocytes (cluster 6 and cluster 8, with marker genes Ly6c2, Vcan, and Fn1); 3 clusters of macrophages (cluster 2, cluster 4, and cluster 9, with marker genes C1qa and Mrc1); 2 clusters of DC cells (cluster 5 and cluster 13, with marker genes Ccr7, Mgl2, Ccl22 Cd209a, and Fscn1), 1 cluster of NK cells (cluster 14, with marker genes Cd3d-, Xcl1, and Ncr1); 1 cluster of T cells (cluster 7, with the main marker genes Cd3d, Cd3e, Cd3g, and Trac); 1 cluster of mast cells (cluster 16, with the main marker genes Ms4a2, Cpa3, Gata2, and Tpsb2); 1 cluster of fibroblasts (cluster 17, with the main marker genes Col1a1 and Dcn); and 1 cluster of cells not previously described (cluster 11), with the main marker genes Acp5, Ctsk, Mmp9, Atp6V0d2, which are noted in the literature as marker genes for osteoclasts (supplementary 2, Table 1).

**Table 1:** Summary of Major Cell Types in the Wounds Healing Process

Cell cluster	No.of cells	Marker genes	Transcription Factors	Major function
Neutrophil Cluster 0	5507	Cxcr2,Csfr3,Il1r2 Lrg1	Cebpd,Egr1,Egr2	inflammatory response, response to lipopolysaccharide, apoptotic process, cytokine-mediated signaling pathway, positive regulation of endothelial cell proliferation, positive regulation of angiogenesis
Neutrophil Cluster 1	4773	Gm5483,IL1f9,Ccl3, Acod1,Stfa211	Cebpd,Egr1,Egr2	inflammatory response, response to lipopolysaccharide, neutrophil chemotaxis, immune response, cytokine-mediated signaling pathway, positive regulation of cytokine production
Neutrophil Cluster3	2899	Retnlg,Ngp,Lcn2, Wfdc21,Mmp8	Cebpd,Egr1,Egr2	inflammatory response, immune system process myeloid dendritic cell chemotaxis, negative regulation of lymphangiogenesis
Neutrophil Cluster12	594	Rsad2,Ifit1,Ifit3, Isg20,Slfn1	Cebpd,Egr1,Egr2	innate immune response, response to virus, defense response to virus
Macrophage Cluster 2	3581	Cd74,C1qb,Ctss, Apoe,Csf1r	Atf3,Irf7,Klf4,Spic	antigen processing and presentation of exogenous peptide antigen via MHC class II, immune system process, immune response, apoptotic cell clearance
Macrophage Cluster 4	2793	Ccl8,C1qc,Selenop, Mrc1,Cbr2,Igf1,Cd163	Atf3,Egr1,Egr2,Irf7, Jun, Klf4,Mafb,Nr1d1,Spic	inflammatory response, endocytosis, response to drug,angiogenesis, positive regulation of smooth muscle cell proliferation, positive regulation of cell proliferation
Macrophage Cluster 9	1587	Gpnmb,Pf4,Lpl,Cd36,Fabp5	Atf3,Egr2,Irf7,Klf4, Mafb,Nr1d1,Spic	inflammatory response, lipid metabolic process, response to oxidative stress, response to oxidative stress
Monocyte Cluster 6	2370	Arg1,Pdpn,Ccl2,Cxcl1,Fn1	Atf3,Egr2,Irf7, Klf4,Prdm1,Spic	Angiogenesis, Inflammatory response, immune response
Monocyte Cluster 8	2140	Plac8,Chil3,Vcan,Ly6c2,Lyz2	Atf3,Egr2,Irf7, Klf4,Spic	Translation, monocyte chemotaxis, positive regulation of T cell activation
Dendritic cell Cluster 5	2497	H2-Ab1,H2-Aa,H2-Eb1, cd209a,H2-DMa,H2-Dmb1	Irf7,Atf3,Nr4a2,Spib	ribosomal small subunit biogenesis, antigen processing and presentation of peptide or polysaccharide antigen via MHC class IIc, regulation of T cell proliferation
Dendritic cell Cluster 13	563	Fscn1,Ccl22,Tbc1d4, Ccr7,Tmem123,Cacnb3	Atf5,Ehf,Spib	Translation, antigen processing and presentation, immune system process
T cells Cluster 7	2148	Icos,Rora,Ets1	Gata3,Rora,Tcf7, Stat4,Crem	T cell activation, T cell differentiation
Osteoclast Cluster 11	829	Ctsk,Acp5,mmp9,Atp6V0d2	Atf3,Irf7,Klf4, Nr4a2	Translation, RNA splicing, mRNA processing, oxidation-reduction process
NK cells Cluster 14	487	Gzma,Ccl5,Il2rb,Xc1l,Nkg7	Eomes,Gata3,Myb, Tcf7	Cytolysis, response to virus, positive regulation of T cell mediated cytotoxicity
Mast Cells Cluster 16	147	Cpa3,Serpinb1a,Tpsab1, Mrgprb1,Gata2	Egr1,Gata2,	positive regulation of mast cell degranulation
Fibroblast Cluster 17	50	Fstl1,Bgn,Col5a1, Col16a1,Mmp2	Ebf1,EGr1,Plagl1, Tbx15,Twist2	cell adhesion, collagen fibril organization, extracellular matrix organization

### F2 scRNA-seq analysis reveals a dynamic immune landscape in STZ-induced diabetic mouse wounds

**Fig. 2:**
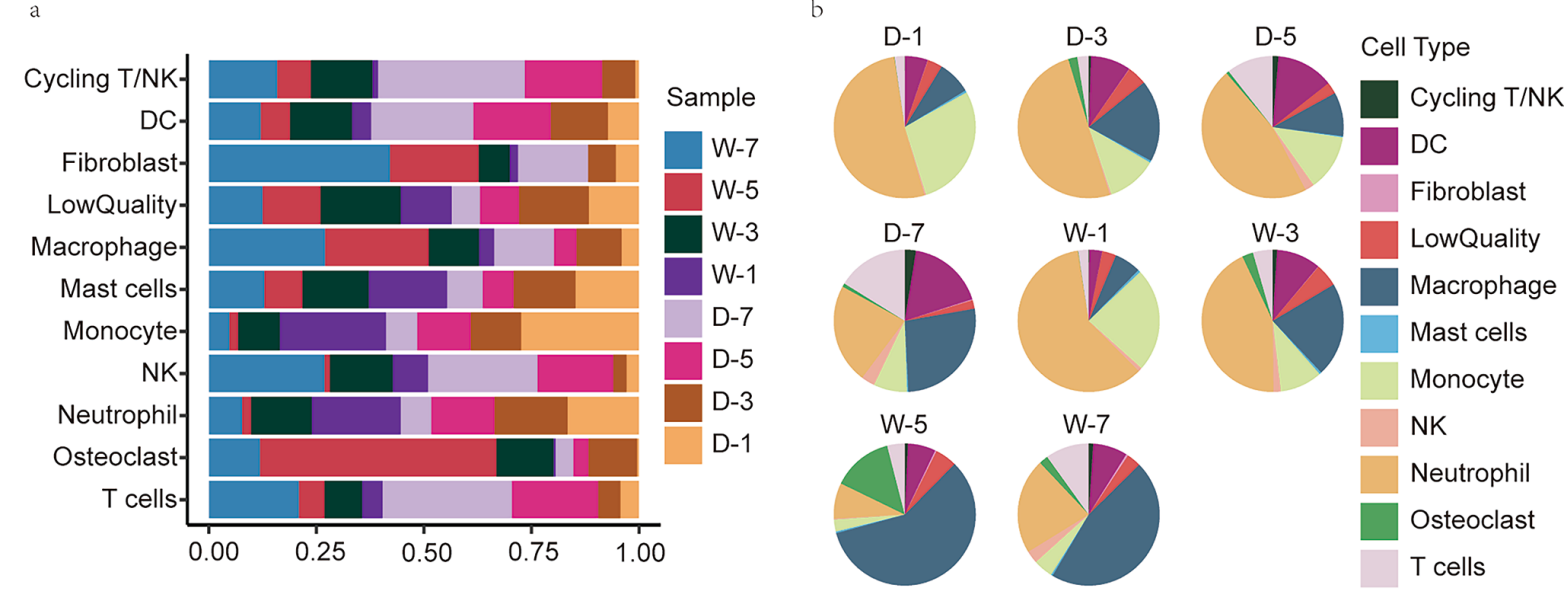
scRNA-seq analysis eveals a dynamic immune landscape in STZ-induced diabetic mouse wounds. **(a)** Stacked bar plots showing the proportion of cells from each sample source among the different cell types (D-1: diabetes group day 1, D-3: diabetes group day 3, D-5: diabetes group day 5, D-7: diabetes group day 7, W-1: Wildtype control group day1, etc.) **(b)** Pie chart plots showing the proportion of various cells in different samples

After obtaining the overall spectrum of immune cells, we further counted the number of each group of immune cells in the two groups of wounds according to different time points and combined it with their gene expression. Exploring the differences in immune status between two groups of wounds healing process and their possible underlying causes (Fig. 2; Table 2, Supplementary 3).

**Table 2:** Summary of Cell Numbers in Differently Sampling Time

Cluster	CellType	Samples and Cell Number							
		D-1	D-3	D-5	D-7	W-1	W-3	W-5	W-7
0	Neutrophil	196	1288	662	643	209	1352	216	941
1	Neutrophil	860	1167	895	127	1038	605	32	49
2	Macrophage	292	440	252	473	141	695	490	798
3	Neutrophil	1085	207	312	15	1191	75	7	7
4	Macrophage	34	207	48	307	29	162	923	1083
5	DC	215	400	421	502	103	405	153	298
6	Monocyte	698	286	206	175	699	103	61	142
7	T cells	106	140	437	566	103	213	125	458
8	Monocyte	611	338	323	92	334	355	24	63
9	Macrophage	28	369	112	163	95	165	420	235
10	LowQuality	157	241	109	68	139	249	157	150
11	Osteoclast	4	114	28	29	4	120	434	96
12	Neutrophil	273	65	40	11	180	18	0	7
13	DC	26	97	117	111	27	75	46	64
14	NK	16	19	86	108	40	80	6	132
15	Cycling T/NK	3	29	54	89	4	48	23	48
16	Mast cells	23	25	10	10	25	24	12	18
17	Fibroblast	3	4	0	7	1	4	10	21

The population of neutrophils that responded earliest to wound healing also had the correspondingly highest proportion of total cell counts, and the proportion decreased over the healing process, but the proportion of neutrophils declined gently on early days 1, 3, and 5 in the diabetic group, whereas a steep decline occurred on day 5 in the control group. The change of subcluster Retnlg + Lcn2 + Wfdc21 + Mmp8 + neutrophils (cluster3) was noteworthy, with the initial cell count in the control group consistent with that of the diabetic group (day1, 1085 vs. 1191) and then dropping rapidly to low levels (day5, 312 vs. 7). Lcn2 promotes neutrophil recruitment and can contribute to inflammation through synergistic Th17 (Hau et al. 2016; Shashidharamurthy et al. 2013). It is also a marker of inflammation associated with obesity and insulin resistance (Wang et al. 2007). The enrichment of MMp8 in chronic inflammation and its ability to degrade the extracellular matrix suggest that this group of neutrophils may be a factor in the chronic healing of diabetic wounds (Diegelmann 2003).

At day1 monocytes were the second most abundant cell type and then began to decline, with the proportion of monocytes on day 5 in the control group decreasing dramatically and being significantly lower than in the diabetic group (1.83% vs. 11.28%). Monocytes were divided into two groups of subclusters, Arg1 + Pdpn + Ccl2 + Cxcl1 + Fn1 + monocytes (cluster6). The functions of cluster 6 c include Angiogenesis in addition to Inflammatory response, immune response. Although Arg1 has been reported to be elevated in ischaemic chronic wounds (Roy et al. 2009), the initial cluster6 cells counts we observed in both groups of wounds were consistent, so the differential decrease in this group of monocytes may not be due to a compensatory effect but may be due to a blocked conversion of monocytes to macrophages in diabetic wounds. In contrast, the other group of Plac8 + chil3 + Vcan + Ly6c2^hi^, CCR2^hi^ monocytes (Cluster8) was consistent with the inflammatory monocytes reported previously (Shi and Pamer 2011). The number of cells in this group was significantly higher in the diabetic group than in the control group at the beginning (day1, 611 vs. 334) and reversed at the end (day5, 323 vs. 24). These results suggest that excessive inflammation in diabetic wounds in terms of monocytes may be the result of a combination of pro-inflammatory monocyte retention and impaired monocyte-macrophage transformation.

DC cells regulate and activate endogenous and adaptive immunity, further activating T cells through antigen presentation. We observed a gradual increase in the proportion of Dc cells in diabetic wounds from day1-day7, with a regression in the number of cells in the control wounds group from day5-day7. In addition to the classical Dc cells expressing major histocompatibility complex class II (cluster5, H2-Ab1 + H2-Aa + H2-Eb1+) there was also a group of cells expressing the Fscn1, Ccl22, Tbc1d4, Ccr7 marker gene, migration-related (CCR7, FSCN1), and encoding chemokine ligands (CCL22) suggest the function of recruit immune cells, mostly Tregs (Peng et al. 2022).The proportion of T cells (cluster7, Icos + Rora + Ets1+) followed the same trend as that of Dc cells.

Day5 was the cut-off point for the change in the proportion of numerous cells in both wounds group, and the differences in the immune profile between wounds in terms of the number and proportion of cells accumulated from day1 to day3, after which the differences in the degree of inflammation between the control and diabetic wounds groups became highly significant. The precise timing of interventions selected for different immune cell populations appears to be important in promoting diabetic wound healing.

### F3 Gene expression characteristics and biological function analysis of cluster 11 and the gene expression differences compared with other macrophages

**Fig. 3:**
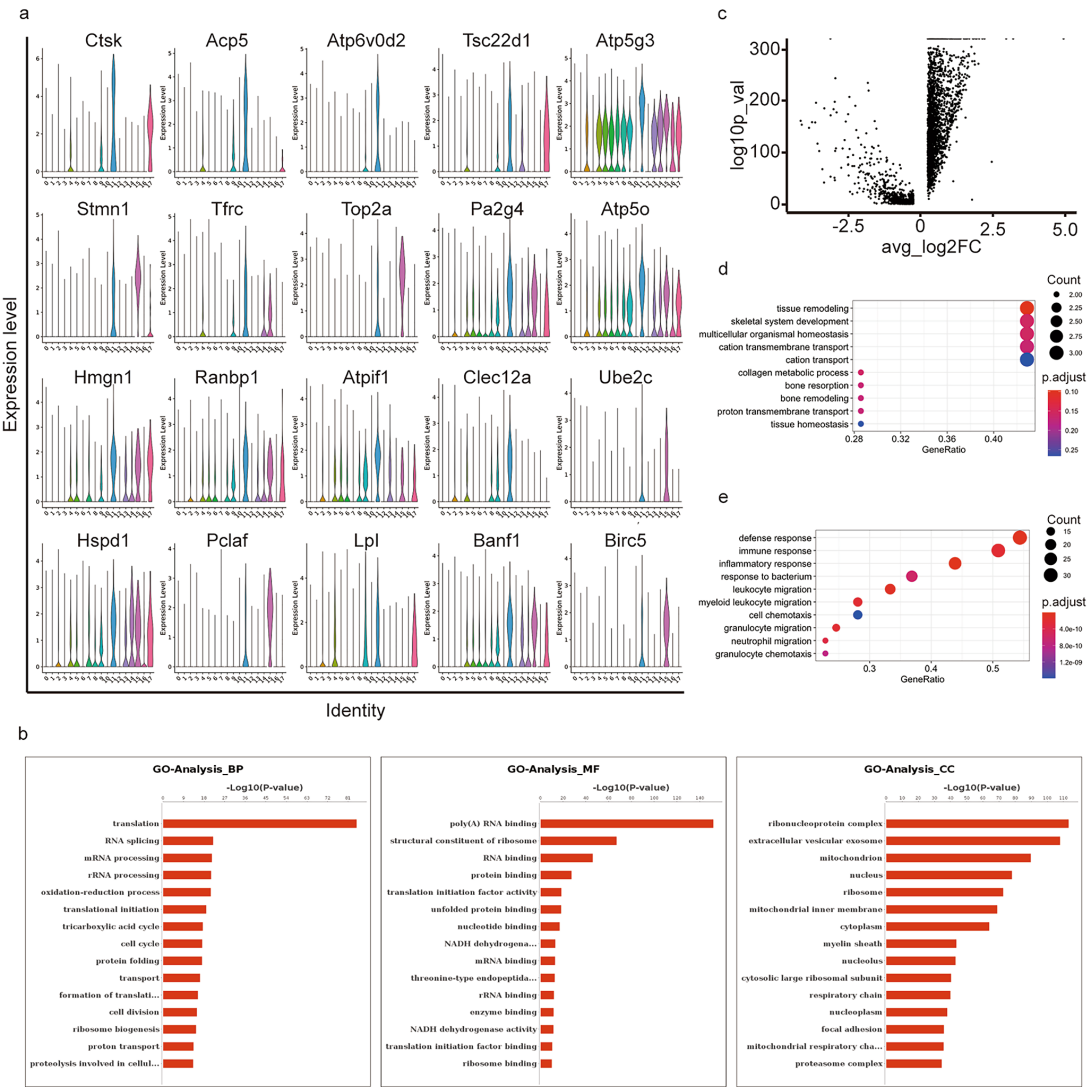
Gene expression characteristics and biological function analysis of cluster 11 and the gene expression differences compared with other macrophages. **(a)** Violin plot view cluster11 top 20 marker gene demonstrating overall gene expression. The number of identity is the same of clusters **(b)** GO histogram analysis results of cluster11 marker gene: Biological Process (BP), Molecular Function (MF), Cellular Component (CC). Coordinate axis Y: Go-Term entry name,Coordinate axis X: -log10 (P-Value). Red for significant entries, blue for non-significant entries **(c)** Volcano plot view for the gene expression difference between cluster11 and other macrophages(cluster2,4,9).Coordinate axis Y: -log10(P-Value), axis X:avg_log2FC.X<-1 use pink color as down expression, X > 1 use blue color as up expression **(d, e)** GO analysis up regulated (d) and down regulated gene (e) of cluster11: Biological Process, Coordinate axis Y: Go-Term entry name, Coordinate axis X: Gene Ratio. Colors of the bubble represents P.adjust < 0.05 for significant entries. The size of the bubble indicates the number of genes enriched in this item

The steep increase in the numbers of cells expressing osteoclast marker gene (clusters11) in the control wounds group attracted our attention. To characterize cluster 11 as a specific group of immune cells, we mapped the top 20 marker genes on a violin plot (Fig. 3a) and performed GO functional enrichment analysis of the marker genes. The genes that were highly expressed were the osteoclast-associated genes Ctsk and Acp5; the adipose tissue-associated genes Hmgn1, Ranbp1 and Lpl; and the macrophage-associated genes Tsc22d1 and Banf1. The cycling basal cell-related genes Stmn1, Top2a, Ube2c, Pclaf, and Birc5 suggest that this group of cells may be a previously undescribed type of skin-resident macrophage. The GO functional enrichment analysis results showed that the gene functions were mainly related to translation, RNA splicing, mRNA processing, rRNA processing, oxidation-reduction process, translational initiation tricarboxylic acid cycle, cell cycle, protein folding, transport, etc. (Fig. 3b) We also applied cell cycle analysis, according to G2M.Score, only a small part of cluster 11 cells were enriched for cell cycle gene (Stmn1, Top2a). (Supplementary 4), The self-renewal and proliferation of this small number of cells indicates that this group of cells is actively involved in the healing process and suggests that our data are indicative of the dynamic characteristics of this group of cells during the healing process.

We further compared the gene expression differences between cluster 11 and all other macrophages (cluster 2, cluster 4, and cluster 9). A total of 230 genes were upregulated and 205 genes were downregulated in cluster 11 compared to the other macrophage populations (Fig. 3c). GO enrichment of the differential genes showed that upregulated genes were enriched in tissue remodeling, skeletal system development, multicellular organismal homeostasis, cation transmembrane transport, cation transport, collagen metabolic process, bone resorption, bone remodeling, porton transmembrane transport, and tissue homeostasis (Fig. 3d). Biological functions of the downregulated genes are enriched in defense response, immune response, inflammatory response, response to bacterium, leukocyte migration, myeloid leukocyte migration, cell chemotaxis, granulocyte migration, neutrophil migration, and granulocyte chemotaxis (Fig. 3e). These results suggest that this group of cells is not primarily involved in the inflammatory process, instead may be involved in the wound healing process by balancing tissue homeostasis, tissue remodelling, and collagen metabolism in the extracellular matrix. This also explains the difference in their distribution between the two groups of wounds samples.

### F4 Macrophage gene metabolism pattern analysis and cell-cell contact

**Fig. 4:**
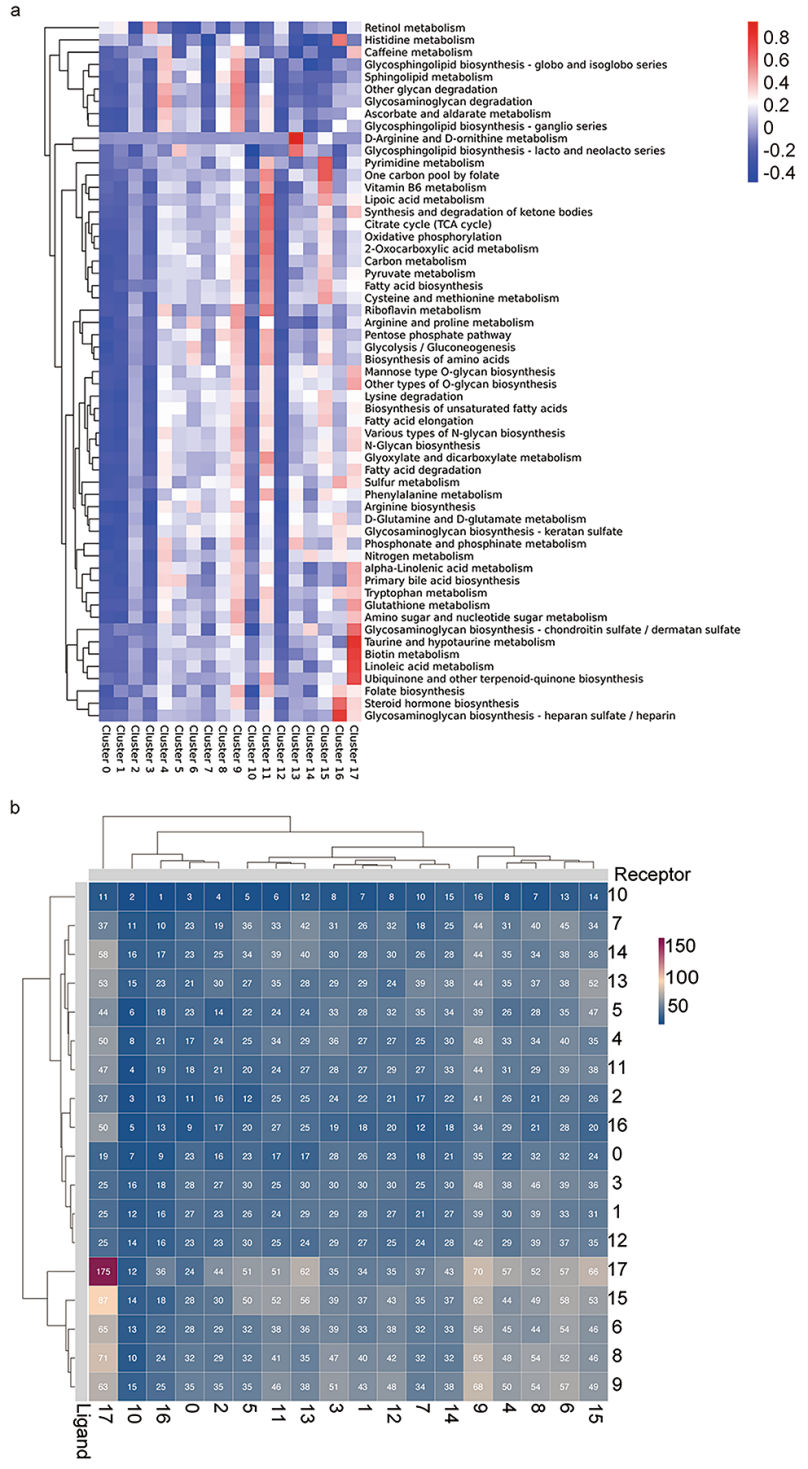
Macrophage gene metabolism pattern analysis and cell-cell contact. **(a)** Heatmap of Qusage Analysis, shows the significance of enrichment between cluster in metabolism gene set.Axis Y: Gene set information,aixs X:clusters. The colour represents the significance of each cluster in each gene set, the closer the colour to red, the more significant it is; the closer the colour to blue, the less significant it is **(b)** Heatmap show number of potential ligand-receptor pairs between immune cell groups predicted by CellphoneDB

We observed that the differentially expressed genes in cluster 11 were enriched in multiple metabolic pathways, and we generated a metabolism heatmap for all cell populations. The gene metabolism patterns of cluster 11 were highly enriched in one-carbon pool by folate, vitamin B6 metabolism, lipoic acid metabolism, synthesis and degradation of ketone bodies, citrate cycle, oxidative phosphorylation, 2-oxocarboxylic acid metabolism, carbon metabolism, pyruvate metabolism, fatty acid biosynthesis, and cysteine and methionine metabolism. Among the remaining macrophage populations, cluster 4 and cluster 9 showed some similarity in gene metabolism patterns and differed significantly from cluster 2. The similarities between cluster 4 and cluster 9 were mainly enriched in caffeine metabolism, glycosphingolipid biosynthesis – globo and isoglobo series, sphingolipid metabolism, other glycan degradation, glycosaminoglycan degradation, ascorbate and aldarate metabolism, and glycosphingolipid biosynthesis – ganglio series (Fig. 4a). Macrophage function is dependent on different metabolic pathways, and the metabolism-related gene set of cluster11 is actively enriched, particularly in the tricarboxylic acid cycle and glycolysis-related genes. Tissue and vascular-related damage and hypoxia during the inflammatory phase have little effect on inflammatory macrophages, which are mainly dependent on glycolysis (Murdoch et al. 2005). The enrichment of anabolic metabolism is one of the key features of the tissue proliferation, repair and remodelling phase, and this population of macrophages, which peaks during the repair phase, achieves its repair-promoting function through active metabolism, while the high glucose environment of diabetic wounds and its induced production of ROS affects the activation and function of aerobic metabolic pathways of macrophages (Rendra et al. 2019). In particular, this population of cells is not active in metabolism with arginine and ornithine compared to the traditionally defined alternative activated macrophages, as a potential target for metabolism-related interventions that may avoid excessive scarring and fibrosis (Liu et al. 2017).

The violin plots for the marker genes expressed in cluster 2, cluster 4, and cluster 9 showed that cluster 4 expressed genes that were similar to those previously defined as “M2 macrophages” (Mrc1 and cd163). Cluster 2 had more pro-inflammatory genes, and the genes cd74, tnsf9, tnsf12, and tnsf12a were highly expressed. Gene expression of Gpnmb, Pf4, Lpl, Cd36, Apoe were found more significant in cluster9 (supplementary 5).

To further characterize cell-cell interactions, we inferred putative cell-cell interactions based on ligand-receptor signaling inferred from our scRNA-seq data using CellPhoneDB. fibroblasts and macrophages showed the most interactions (Fig. 4b). Further visualization of intercellular interactions revealed that hebp1/Fprs ligand-receptor pairs are widespread among neutrophil macrophages and mediate the recruitment of monocytes to play a reparative role (Birkl et al. 2019). The cell-cell contact between cluster11 and neutrophils is supported by Sema4d/ Cd72 (Supplementary 6), and the promotion of cluster11 production by neutrophils may be related to Sema4D inhibition of osteogenic activity and promotion of osteoclastogenesis (Shindo et al. 2022). Cluster2 pro-inflammatory macrophages communicate with monocytes (cluster8), macrophages (cluster4/9) as well as Dc cells (cluster13), fibroblasts (cluster17) via Grn/Sort1 ligand-receptor pairs (supplementary 7), and the multiple involvements of this immunomodulatory mechanism in our cutaneous trabecular immune cells include in Grn/Sort1 regulates the migration and division of fibroblasts for angiogenesis and the recruitment and activity of immune cells during wound repair (Terryn et al. 2021). According to our findings Grn can be used as one of the biological indicators of the intensity of the inflammatory response in skin wounds.

The group of fibroblasts (cluster 17, Pdgfra^high^, Acta2+, CD45+, Col12a1+) we identified while sorting myeloid cells had some similarities to fibroblasts that have been previously reported to be differentiated from trabecular myeloid cells (Haensel et al. 2020). Due to our prior cell sorting based on myeloid markers, fibroblasts were less numerous but showed strong auto cellular communication (Fig. 4b), which we observed in greater numbers in control group, and the expression of secreted cytokines such as VEGF supported their role in repair angiogenesis (supplementary3, 8). Other fibroblast populations could not be located in our samples, so the role of interfibroblast communication could not be clarified, but the role of these myeloid-derived fibroblasts on other myofibroblasts in the wounds was seen in earlier reports(Suga et al., 2014). These myeloid-derived intermediate cells are very easy to miss in the in vitro observation of fibroblasts and single-cell sequencing provides an excellent tool for analysis.

### F5 Differences in the proportion of macrophages over time and the differences in gene expression between the diabetic wound group and the control group

**Fig. 5:**
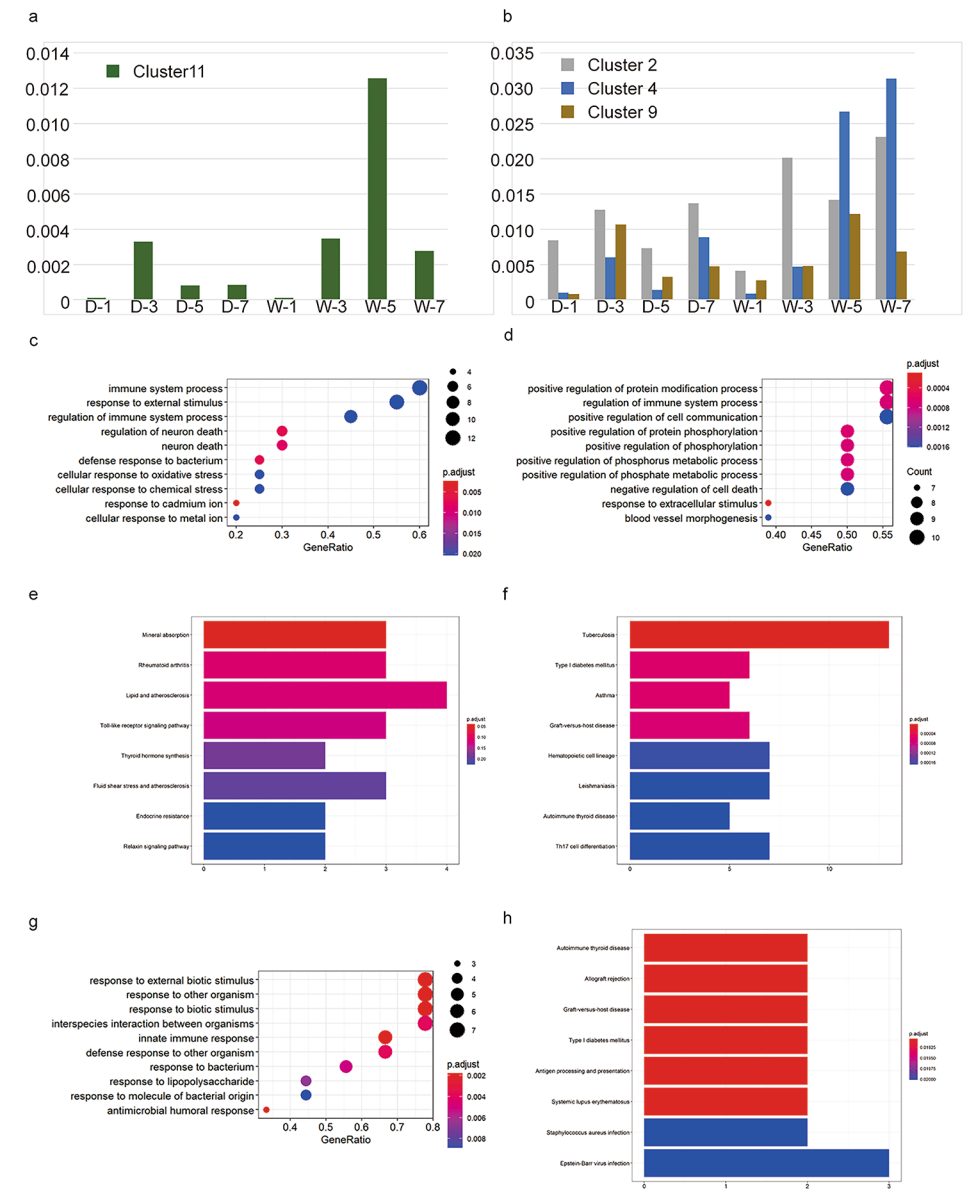
Differences in the proportion of macrophages over time and the differences in gene expression between the diabetic wound group and the control group. **(a)** The proportion of cluster 11 cells in the control group on day 5 and day 7 was much higher than diabetic group. Coordinate axis Y: Proportion of specific cluster of cells in all single cells, Coordinate axis X: cell clusters, group and sampling time. Ex: D-1(Diabetic group -day 1), W-1(Wild type control group-day 1) **(b)** The proportion of cluster 2, cluster 4, and cluster 9 cells in the control group and diabetic group **(c)** GO enrichment analysis of the differential genes and found that the differences in the biological functions between the two groups with respect to cluster 11 on day 3 **(d)** GO enrichment analysis of the downregulated genes in cluster 11 on day 5 in the diabetes group and the biological functions of the differences **(e)** KEGG terms of control and diabetes two groups of cluster 11 cells on day 3 differentially expressed genes **(f)** KEGG terms of the cluster 11 cells on day 7 upregulated genes in the diabetes group **(g)** GO enrichment analysis of the upregulated genes in cluster 4 on day 5 in the diabetic group and the biological functions of the differences **(h)** KEGG enriched differential gene pathways on day 5 of cluster 4

The phenotypic changes and overall proportional changes in macrophages in the two different subgroups are also an important part of our understanding of their mechanisms. Thus, we counted the proportional changes in the macrophage populations in the two experimental groups at different sampling times, and the proportion of the cluster 11 cell population increased in the early stage (day 1–day 3) in both the diabetic wound group and the control group, but unlike the diabetic wound group, the proportion of this cell population in the control group increased consistently (1.26%) on day 5 and was much higher than that in the diabetic wound group (0.08%) and decreased (0.28%) on day 7, but the proportion was still higher than that of the diabetic group (0.08%) (Fig. 5a). The proportion of Cluster 2 cells was higher in diabetic groups on day 1(0.85% versus 0.41%), with similar trends in cell proportions within both groups. After day 3 the proportion of cluster2 cells was higher in the control wound group than in the diabetic wound group (1.42% versus 0.73%). Cluster 4 showed a gradual increase in the proportion of cells in the diabetic wound group, except on day 5. In the control group, however, a much higher increase was observed on day 5 (3.14%) and day 7 (8.09%) than that in the diabetic group. A peak in the proportion of cluster 9 was observed in the diabetic group (1.07%) at an earlier time point (on day 3) than in the control group (1.22% on day 5) (Fig. 5b).

It is well known that the immune environment of the diabetic group differs from that of the control group, so the specific differences in the macrophage population at different time points are of interest to us. In the next step, we performed GO enrichment analysis of the differential genes and found that differences in the biological functions between the two groups with respect to cluster 11 on day 3. The number of cells in the two groups was very similar at day3, and the genes we found to be different included Acod1, Ccl3, Ctsk, vHsap5, Mmp9, and Fos, the functions of these genes were mainly enriched in immune system process, response to external stimulus, regulation of immune system process, regulation of neuron death, defense response to bacterium, and cellular response to oxidative stress (Fig. 5c). Differentially expressed genes CCl3, Hsap5, Mmp9, Fos, Ctsk corresponded to rheumatoid arthritis, lipid and atherosclerosis, and the Toll-like receptor signaling pathway (Fig. 5e). As the wound healing progressed we found a huge difference in the number of this group of cells on day5, the peak number of cluster11 osteoblast-like macrophages. Apoe, Ccl7, Cd36, and Fnip1 genes were downregulated in the diabetic group compared to the control group on day5, the biological functions of the downregulated genes were enriched in positive regulation of protein modification process, positive regulation of cell communication, positive regulation of protein phosphorylation, positive regulation of phosphorus metabolic process, negative regulation of cell death, The downregulation of the Hmox1, Jun, Pf4 gene suggests that this group of cells Capacity of blood vessel morphogenesis has also been reduced (Fig. 5d). Then at day 7, the end of our observation H2-Aa, H2-Ab1, H2-DMa, H2-DMb1, H2-Eb1, Il1b genes were upregulated in the diabetes group corresponded to type I diabetes and Th17 cell differentiation (Fig. 5f).

As for the pro-inflammatory macrophage-cluster2, the expression level of Acod1, Il1a, Mt1, Retnlg, Osal1, Lyz1, Saa3, CD163, was different in day 3, functions of these genes are enriched in response to stress, defence response, response to external stimulus, response to external biotic stimulus, response to other organism, response to biotic stimulus, interspecies interaction between organisms, response to bacterium, immune response, inflammatory response, and defence response to other organism. This enhanced immune response capacity is in line with our expectations. In parallel to the downregulated gene of pro-inflammatory macrophages in the diabetic group, mainly Lpl, which functions in cytokine production and regulation of cytokine production and with the healing process, in day5 we observed a decrease in Lpl, Cd36, Lipa genes which could suggest the possible involvement of cholesterol metabolism pathway in the progression of inflammatory cells. Inflammatory pathways suppress cholesterol metabolism and reverse cholesterol transport (RCT) which in turn enhances inflammatory responses, previously reported mainly in atherosclerosis-related studies, a similar mechanism can now be considered in diabetic skin damage (Groenen et al. 2021; Westerterp et al. 2018; Yvan-Charvet et al. 2008) (Supplementary 9).

On day 1 the cluster 4 macrophages differentially expressed genes Lgals3, Bcl2, Sod2, Hsph1 biological functions of these genes were enriched in positive regulation of developmental process, negative regulation of apoptotic signalling pathway, Ccl3, Tlr2, Tnfaip3 genes were enriched in interleukin-1 beta production, and interleukin-1 production. On day 3 differential genes Acod1, Cd209b, Cd209d, Clec4e were enriched in response to external stimulus, defence response, interspecies interaction between organisms, response to external biotic stimulus, response to other organism, andCd163, Cfh, Chil3 inflammatory response (Supplementary 10). The biological functions of the upregulated genes at day 5 in the diabetic group Acod1, H2-Aa, H2-Eb1, S100a8, A100a9, Slpi were enriched in response to external biotic stimulus, response to other organism, response to biotic stimulus, interspecies interaction between organisms, innate immune response, defense response to other organism, and response to lipopolysaccharide (Fig. 5g). The KEGG enriched differential gene H2-Aa, H2-Eb1 on day 5 were autoimmune thyroid disease, allograft rejection, graft-versus-host disease, type 1 diabetes mellitus, antigen processing and presentation, systemic lupus erythematosus, Staphylococcus aureus infection, and viral myocarditis pathways (Fig. 5h).

The downregulated genes in cluster 9 in the diabetes group on day 1 including Arg1, Cxcl3, Plac8, Saa3 were enriched in response to external biotic stimulus, response to other organism, response to biotic stimulus, defence response (Supplementary 11). The downregulated genes at day 3 Ccr2, Egr1, Fos, and Jun were functionally enriched in tissue development, leukocyte differentiation, blood vessel development, vasculature development, cellular response to growth factor stimulus, and response to growth factor positive regulation of endothelial cell proliferation. The upregulated gene on day 5  including Ccl3, S100a8, S100a9, and Tnbs1, their functions were enriched in positive regulation of response to external stimulus, regulation of hydrolase activity, granulocyte chemotaxis, regulation of peptidase activity, granulocyte migration, myeloid leukocyte migration, and leukocyte chemotaxis (Supplementary 11). On day 7 the downregulated genes Egr1,Fos, and Jun were enriched in biological functions including response to abiotic stimulus, cellular response to stress, positive regulation of pri-miRNA transcription by RNA polymerase II, and positive regulation of neuron death (Supplementary 11).

The KEGG analysis showed that the downregulated genes at day 3 Cracr2b, Nr4a1, Fos, Jun, Vegfa were involved in the MAPK signalling pathway, Nr4a1, Fos, Jun were involved in the relaxin signalling pathway, chemical carcinogenesis-receptor activation, and rheumatoid arthritis. The downregulated genes on day 7 Fos, Hspa1a, Hspa1b, and Jun were enriched in the Estrogen signalling pathway, measles, MAPK signalling pathway, lipid and atherosclerosis, prion disease, human T-cell leukaemia virus 1 infection, endocrine resistance, and antigen processing and presentation (Supplementary 11). Taken together these suggest that the MAPK pathway may play a regulatory role in the proliferation and differentiation of this group of macrophage cells.

### F6 Pseudotime analysis of macrophage populations and analysis of the differential gene expression patterns in the developmental branches

**Fig. 6:**
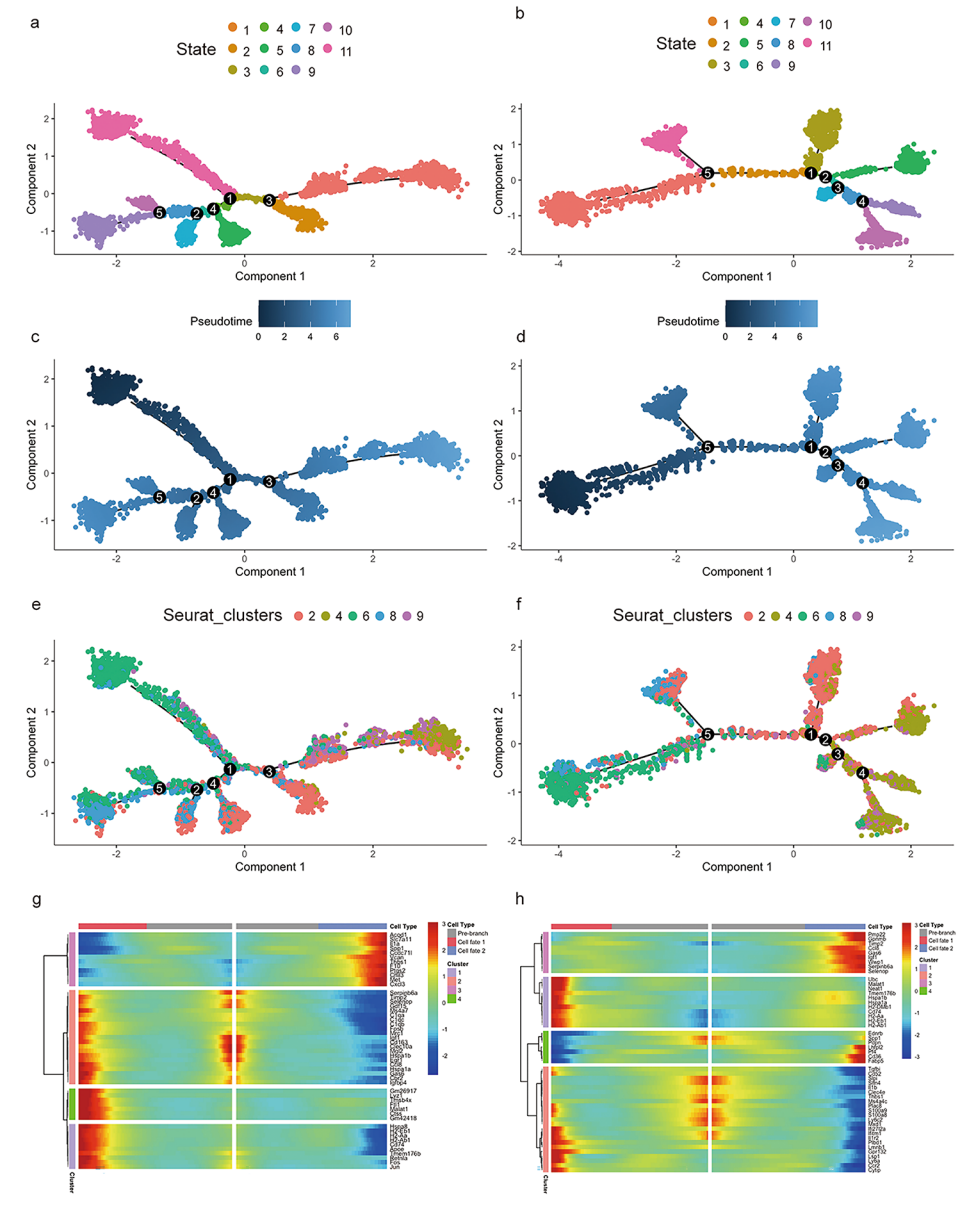
Pseudotime analysis of macrophage populations and analysis of the differential gene expression patterns in the developmental branches. **(a, b)** Reconstruction of the monocyte/macrophage trajectory as 11 state. Diabetes group (a), Control group (b). **(c, d)** Reconstruction of the monocyte/macrophage trajectory in a pseudotime manner. Diabetes group (c), Control group (d). **(e, f)** Reconstruction of the monocyte/macrophage trajectory in cell clusters. Diabetes group (e), Control group (f). **(g, h)** Heatmap of branch 3 gene in diabetes group (g), branch 1 gene in control group (h), the pre-branch in the middle represents all the cells from the branch point to the root cell. Cell fate 1 corresponds to the state with small id while cell fate 2 corresponds to state with bigger id

Monocytes can differentiate into macrophages during the immune process, and macrophages have rich phenotypic diversity and perform different functions at different times during wound healing. We performed a chronological analysis of the observed mononuclear macrophage population and the cells in the diabetic and control wound groups could be classified into 11 states (Fig. 6a and b). According to the pseudotime analysis (Fig. 6c and d), cluster 6 and cluster 8 were predominantly found in the early states, followed by cluster 2, and cluster 4 and cluster 9 were found in large numbers at later time points. In the diabetic wound group, a large number of cluster 4 cells were observed in only one state, whereas in the control group, cluster 4 cell aggregates were observed in several states. In contrast, in the diabetic group, cluster 2 was observed within multiple stages of differentiation (Fig. 6e and f). This finding suggests that within the diabetic group, cell differentiation was more towards cluster 2, whereas in the control group, more branches were differentiated into cluster 4, and the greatest number of cluster 4 aggregates could be seen in the diabetic group with a branch point of 3 compared to 1 in the control group, leading us to more closely analyze the differential gene expression patterns of the two trajectory branches.

In branch 1 of the control group and branch 3 of the diabetic group, a pattern of differential expression consisting of the grouping of genes with a reduction in the differentiation pathway towards cluster 4 and an elevation of the differentiation pathway towards cluster 2 can be observed, with such a pattern seen in branch3 of the diabetic group for Acod1, Slc7a11, il1a, spp1, Ccdc71l, Tnbs1, F10, Ptgs2, Chil3, Met, and Cxcl3 (Fig. 6g). In the control group, there were Tgfbi, cd52, plac8, Ifi2712a, plbd1, lmnb1, gpr132, lsp1, ly6a, ccr2, and cytip in branch1 (Fig. 6h). No crossover genes were found, suggesting that the polarization patterns of macrophages in the control and diabetic group may be quite different.

## Discussion

Using single-cell sequencing unbiased analysis, we described immune cell populations within the wound-associated cells of STZ-induced diabetic and wildtype control mice and defined a population of macrophages expressing osteoclast cell marker genes. The proportion of cd45 + immune cells within the skin was also found to vary between populations at different time points. We proximally analysed the differences in gene expression in macrophage populations at different sampling points and found evidence of temporal variation in the effects of immune dysfunction on wound healing in diabetic mice. The differences in the developmental differentiation trajectory of mononuclear macrophages were also analysed to describe specific differential genes at the macrophage differentiation and polarization branching points.

Our wound-associated macrophage profile yielded three classes of macrophages, with cluster 2 macrophages expressing the cd74, tnfsf9, tnfsf12, and tnfrsf12a genes, suggesting their potential for proinflammatory function. Cd74 is a high affinity receptor on the cell membrane that binds macrophage migration inhibitory factor (MIF) (Su et al. 2017).The interaction of MIF with CD74 can occur at an early stage as a manifestation of the cellular response to injury. CD74 is involved in numerous inflammatory-related disease processes, and recent studies in inflammatory bowel disease (IBD) have shown a strong association between CD74 polymorphisms and the failure of anti-TNF therapy in patients with ulcerative colitis (Yoon et al. 2017). In a mouse model of experimental ischaemia-reperfusion injury, renal tubular injury was more severe in MIF, MIF-2 and CD74 knockout mice than in wild-type control mice(Ochi et al., 2017), and the autoimmune disease systemic lupus erythematosus (SLE) can cause renal inflammation known as lupus nephritis (Almaani et al. 2017). In mouse models of SLE, researchers have observed elevated levels of CD74 expression in B lymphocytes, and elevated MIF has been demonstrated in lupus-prone strains of mice. Inhibition of MIF and knockdown of CD74 protect against glomerulonephritis in lupus-susceptible mice (Lapter et al. 2011; Zhou et al. 2017). CD74 has been less well reported in skin tissue injury, but there is previous evidence in early animal skin injury models that MIF-CD74 promotes the proliferation and migration of keratinocytes at the trabecular margin (Abe et al. 2000). In our experiments, KEGG pathway enrichment analysis suggested that the difference in the LPL gene expression between the wound groups at day 5 was related to the cholesterol metabolic pathway, perhaps leading to a greater response to inflammation in the diabetic wound group than in the control group. Tests of macrophage depletion in wounds at different time points have also shown that early depletion of macrophage populations significantly contributed to delayed wound healing but could attenuate late scar formation (Lucas et al. 2010). This finding suggests that CD74 + immune cells may have different roles within different tissues and in different immune settings. In our present results, CD74^high^ macrophages were observed in the early stages in both groups of samples, with the control group having lower responsiveness than the diabetic group on the first day and a higher overall proportion in the control group at the later stages. This group of macrophages was also observed to express Tgfbi. As CD74^high^ macrophages appear at an early stage as a high proportion of the macrophages, it remains to be investigated whether modulation of CD74 expression can influence scar formation.

Cluster 4 cells expressed the mrc1-cd206, il-10, cd163 and cbr2 genes, with gene expression patterns similar to those traditionally described for M2 macrophages, while cluster 4 cells also expressed the F13a1, lyve1, and gas6 resident-like macrophage genes (Beckers et al. 2017; Ensan et al. 2016). Notably, when comparing the differences in gene expression between cluster 4 cells from the diabetic and control wounds, the diabetic group was found to express higher levels of Lyve1, while the control group expressed higher levels of ccr2, suggesting that this group of cells with the M2 cell gene signature may have origins in both monocyte recruitment and resident macrophages (Cochain et al. 2018). In our experiments, the distribution of M2-like macrophages in both groups was much higher in the control group than in the diabetic group, suggesting that the control group included resident macrophages and more differentiated monocytes recruited from the blood, whereas the M2-like macrophages in the diabetic group were more dependent on their own resident macrophages. A comparative analysis of the gene differences between the two groups at different time points showed that the expression of il-1 was promoted at an early stage in the diabetic group, and later, the diabetic group had a higher level of inflammatory response and defence function than the control group. In contrast, in the control group, the differential gene expression in the early phase was mainly enriched in the functions of proliferation and apoptosis regulation. Higher cd163 expression was observed in the diabetic group than in the control group at both day 3 and day 7. It has also been suggested that sCD163 is higher in type 2 diabetes patients than in healthy individuals (Semnani-Azad et al. 2021). These results may suggest that the elevated feedback appearance of cd163 as an anti-inflammatory gene in macrophages is one of the protective mechanisms promoting wound repair in the diabetic group. The KEGG pathway analysis of the day 5 differential genes showed enrichment in type 1 diabetes and several pathways associated with autoimmune diseases in both groups, suggesting that immune disorders in diabetic patients are more prevalent in this group of macrophages.

Cluster 9 cells are highly similar to cluster 4 in their M2-like macrophage gene expression profile, with the difference being that cluster 9 is highly Gpnmb-expressing, and in tumor-related studies, the tumor-promoting role of myeloid cells is associated with Gpnmb, which can promote cancer cell survival, cancer stem cell expansion and metastatic phenotype acquisition via IL-33(Liguori et al. 2021). This ability to promote stemness was transduced in skin injury to promote stem cell proliferation and repair capacity in the skin, and transplantation of GPNMB-expressing macrophages improved skin healing in GPNMB-mutant mice. Furthermore, topical treatment with recombinant GPNMB restored mesenchymal stem cell recruitment, prompted polarization of wound macrophages towards anti-inflammatory M2 macrophages, and accelerated wound closure in diabetic skin (Yu et al. 2018). It remains to be determined whether this group of cells appearing in a higher abundance at the peak of the proportion of diabetic wounds (day 3) but at a lower level than that in the control group afterward is the result of some inflammatory influence that hinders the proliferation and expression of this group of cells in the mid-term. Our results show that among the enriched GO biological functions of the differentially expressed genes in cluster 9 on day 3, the functions of the downregulated genes are associated with tissue repair, angiogenesis and development and cellular response to growth factors. It has been demonstrated that Junb knockout mice can develop normally, but a lack of Junb under wound conditions results in excessive epidermal skin proliferation and a delayed inflammatory disorder remodelling phase (Florin et al. 2006). Nr4a1, a monocyte transition gene, has been shown to have an important role in genetic models and in the differentiation of monocytes/macrophages in the mouse intestine (Honda et al. 2020). According to the differential analysis, the elevated Gpnmb-high macrophage Tgfbi, IL10, and CD163 expression in the diabetic wound group on day 5 and day 7 may be a result of a delayed repair phase compared to the control group.

Osteoclast macrophages (cluster 11) are an interesting group of cells with high expression of the osteoclast-associated genes Ctsk and Acp5; the adipose tissue-associated genes Hmgn1, Ranbp1, and Lpl; the macrophage-associated genes Tsc22d1 and Banf1; and the cycling basal cell-associated genes Stmn1, Top2a, Ube2c, Pclaf, and Birc5. Under physiological conditions, macrophages and osteoclasts are part of the outcome of monocyte differentiation, and the main determinants of osteoclast production are the relative concentrations of CSF-1, RANKL, and osteoprotegerin (OPG; TNF receptor superfamily member 11B) (Teitelbaum and Ross, 2003; Walsh et al. 2006). In vitro stimulation of RAW264.7 macrophages with RANKL can result in osteoclasts (Song et al. 2019; Zheng et al. 2020). Advanced glycation end products (AGEs) in the diabetic state can affect the expression of bone metabolism proteins (Asadipooya and Uy 2019). Increased reactive oxygen species in diabetes can also affect the balance between osteoclasts and osteoblasts, leading to osteoporosis (Loi et al. 2016). Chronic inflammatory skin diseases such as atopic dermatitis (AD) and psoriasis vulgaris (Pso) are associated with osteoporosis (Shaheen and Silverberg, 2019; Wu et al. 2017). Secondary osteoporosis occurs in a transgenic model of spontaneous dermatitis(Mizutani et al., 2020). All of this evidence suggests that an increase in osteoclasts occurs in response to a strong inflammatory response and stimulation by proinflammatory factors. However, in our study, the proportion of macrophages with osteoclast markers was lower in diabetic mouse wounds with enhanced inflammatory responses, suggesting that, unlike the mechanism of cutaneous inflammation leading to arthritis and osteoporosis, this may be a localized form of cell differentiation specific to the skin.

Human bone, epidermis and hair are all lifelong renewable tissues, and perhaps due to this similarity, except for the role of NF-κB receptor activator (RANK) in osteogenesis and resorption, mice lacking RANK ligand (RANKL) are unable to initiate a new growth phase of the hair cycle and display stalled epidermal homeostasis. RANKL can be expressed in the skin by activated interfollicular epidermis (Barbaroux et al. 2008), and RANK-RANKL regulates hair renewal and epidermal homeostasis and provides a communication channel between these two activities (Duheron et al. 2011). This regulatory ligand for long-term self-renewal may be responsible for the generation of our group of osteoclast-like macrophages. The metabolic pathways and differential gene enrichment results of this group of cells also suggest that they have a strong biosynthetic and metabolic capacity and that the response of this group is stronger in control wounds, suggesting that perhaps this class of skin-specific macrophages contributes to skin homeostasis and repair after damage.

There are still some limitations to this study. The time points chosen for this study are based on previous studies and animal studies and are still somewhat intermittent, as there may be specific peaks in immune cell changes and microenvironmental regulation that do not necessarily occur at the times we chose. The study was conducted at the transcriptional level only and did not include other factors that may affect macrophage function. Further validation of histological level staining for specific cells and the use of knockout animals are needed to better explore and demonstrate the immune-related mechanisms of diabetic wound healing.

These macrophages vary in type and temporal characteristics, and the classical definitions describing the markers and classifications of classically activated macrophages and alternative activated macrophages, or M1 and M2 cells, do not seem to match the descriptions exactly. These results suggest that in vitro studies of immune cells with a single factor and a small number of markers may not accurately model the in vivo environment, especially when analysing the differences in their temporal patterns. More precise gene profiling of cells in vivo and improvements in the way in which they are tracked in vivo may better enable us to identify new immune regulatory mechanisms and therapeutic targets.

## Conclusion

In summary, we characterized the genetic profile of macrophage populations in wounds of diabetic and wildtype control mice by single-cell sequencing and identified a population of macrophages with osteoclast-like gene expression, presumably associated with skin renewal and repair responses. We described the genetic differences between the different cell populations in the wounds of the two groups according to chronological order, providing a closer look into specific processes. Some targets are important as reference makers for macrophages in the field of wound repair.

## Electronic supplementary material

Below is the link to the electronic supplementary material.

Supplementary Material 1

Supplementary Material 2

## Data Availability

All sequencing data were submitted to the BioProject of National Center for Biotechnology Information (NCBI), GEO Submission (GSE186821). Code for data analyses is described in the “Methods” and is available from https://github.com/Lioxuxu/majiaxu.

## Abbreviations

STZ: Streptozocin
FBS: Fetal bovine serum
DPBS: Dulbecco’s Phosphate Buffered Saline
GO: Gene Ontology
KEGG: Kyoto Encyclopedia of Genes and Genomes
