# Supplementary material for: Single-cell RNA-Seq analysis of diabetic wound macrophages in STZ-induced mice
Source: J Cell Commun Signal. 2022 Nov 29;17(1):103–20. doi: 10.1007/s12079-022-00707-w (PMC10030741; doi:10.1007/s12079-022-00707-w)

Supplementary 1: t-SNE visualization of the gene expression/percent.mito/nCount_RNA of each cell.


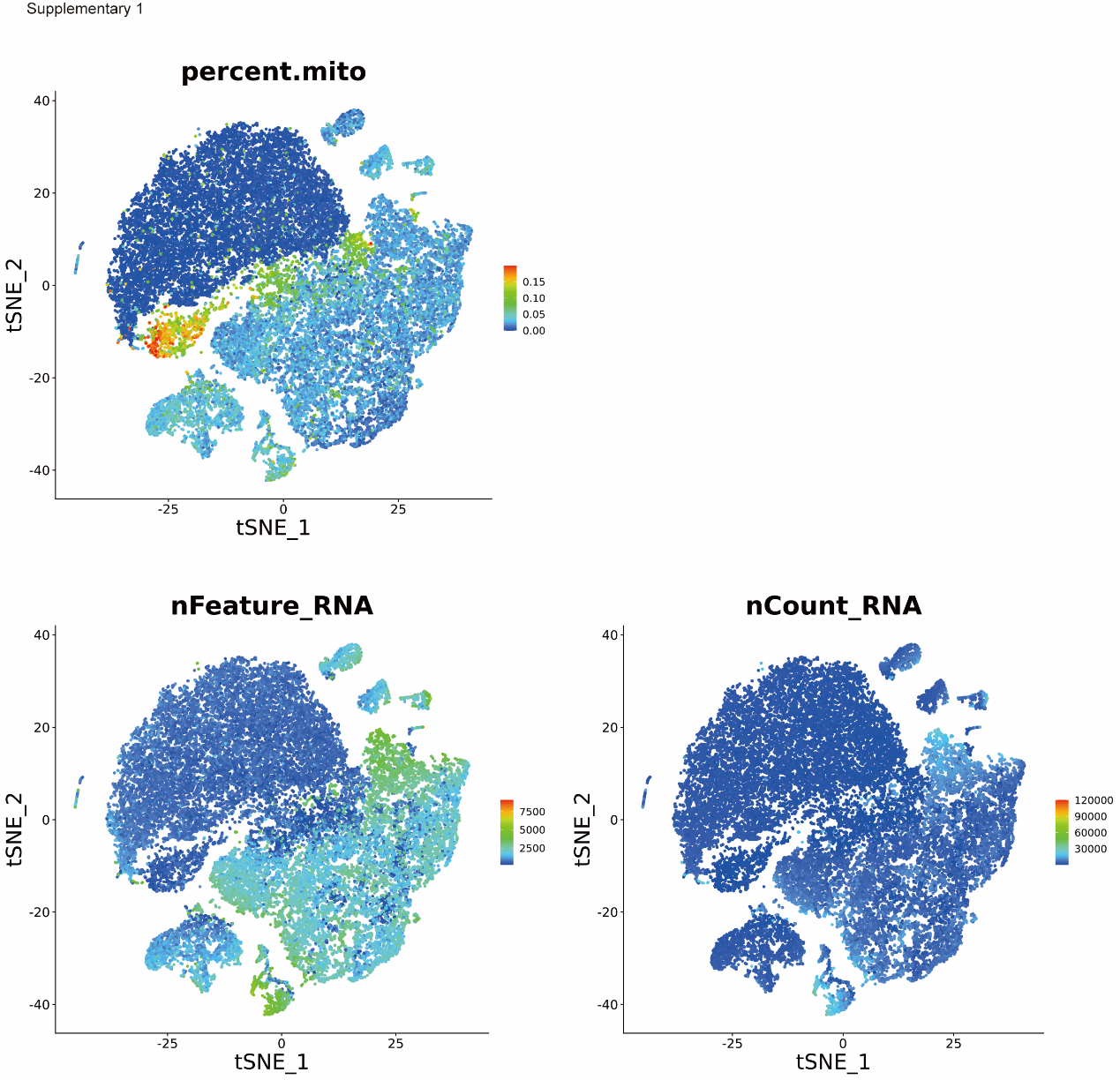


Supplementary 2: Distribution of the top 20 genes in cluster 11 in all cells. Red color indicate high gene expression.


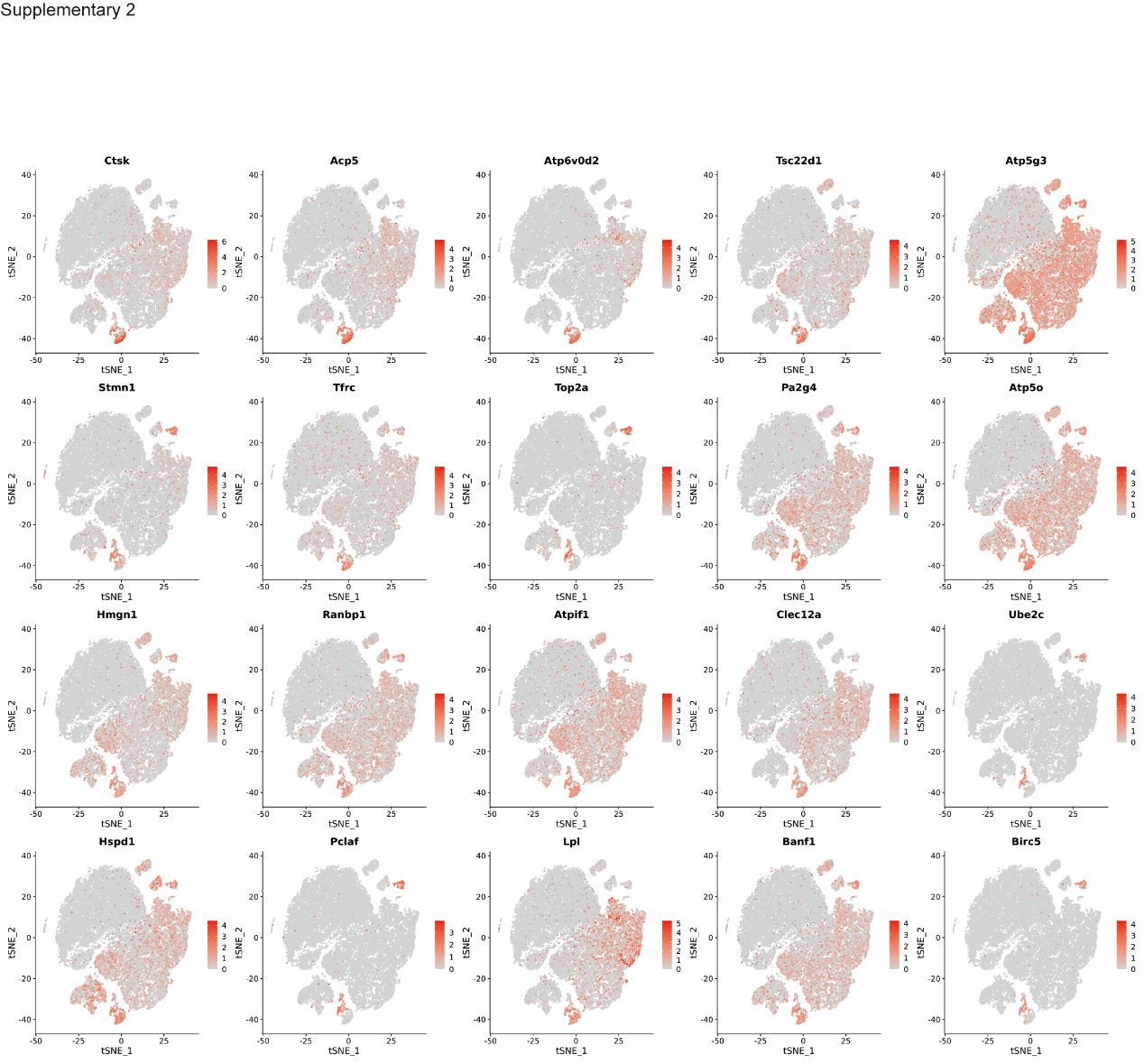


Supplementary 3: An Excle contained all marker genes of all clusters.

Supplementary 4: u-MAP visualization G2M.Score of cluster11 cells and u-MAP visualization distribution of the cell cycle marker gene.


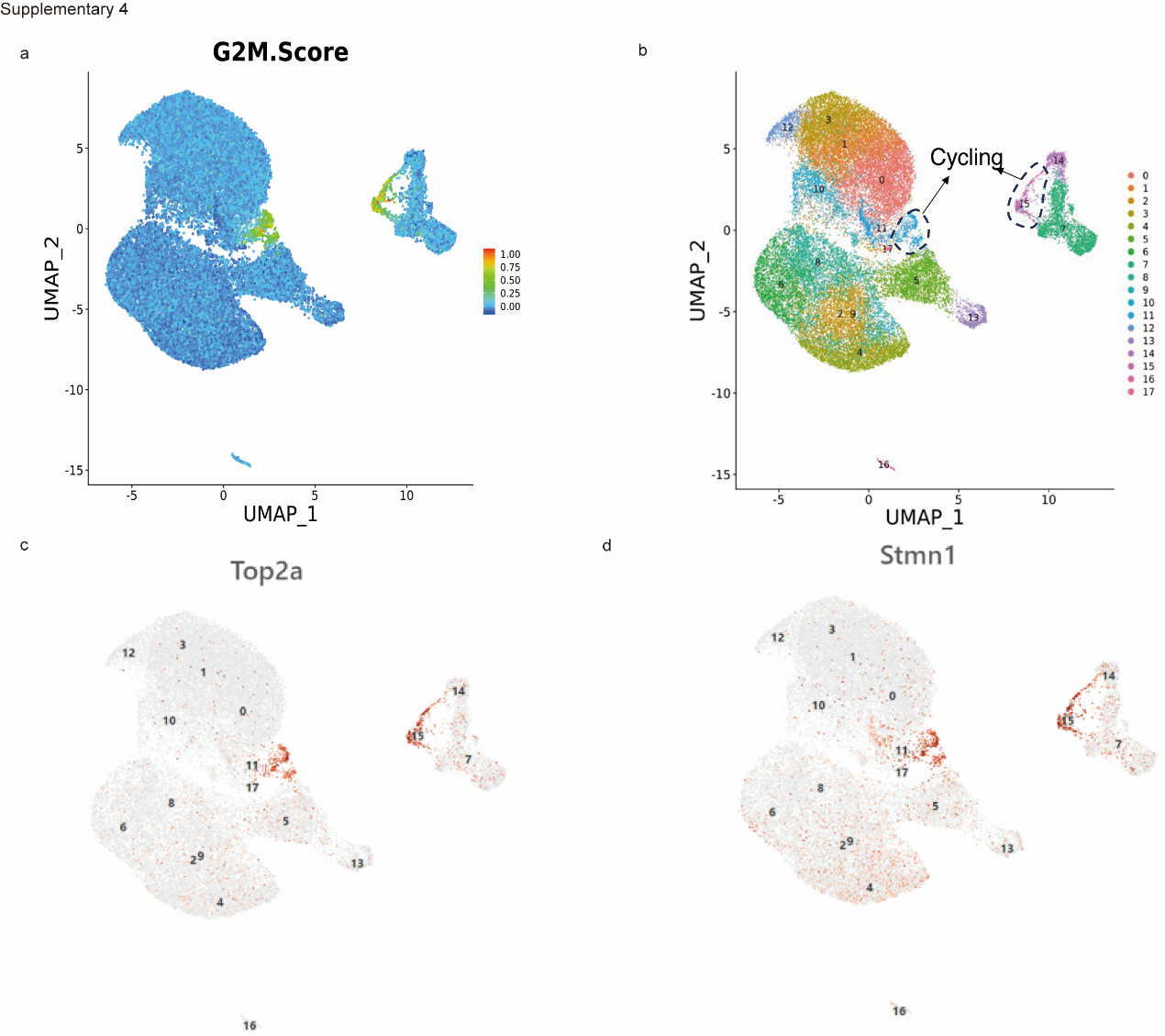


Supplementary 5: Violin plot view top 20 marker gene demonstrating overall gene expression of cluster2, cluster4, and cluster9.


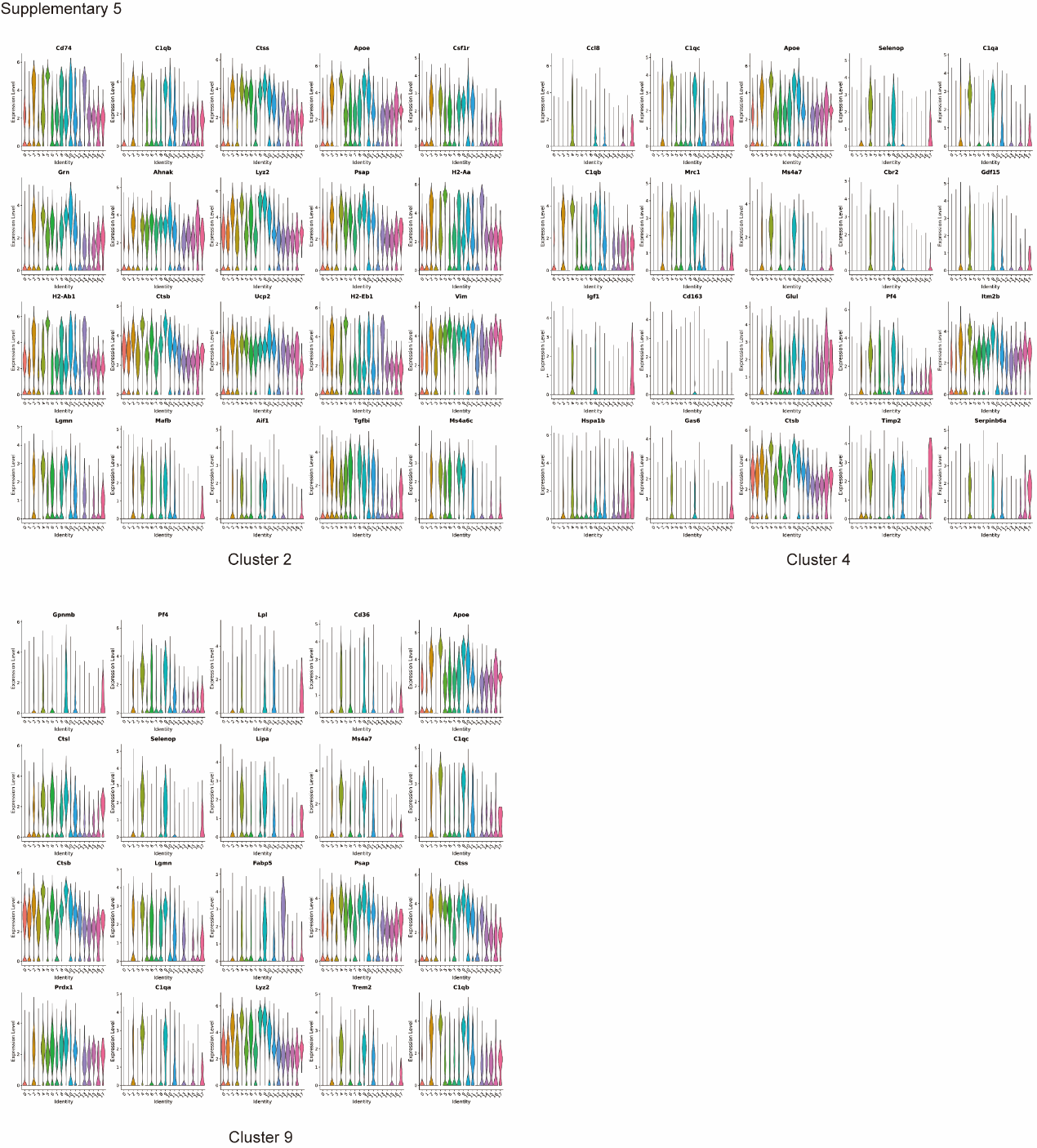


Supplementary 6: Dot plot showed selected ligand-receptor interactions between cluster11 and immune cells.


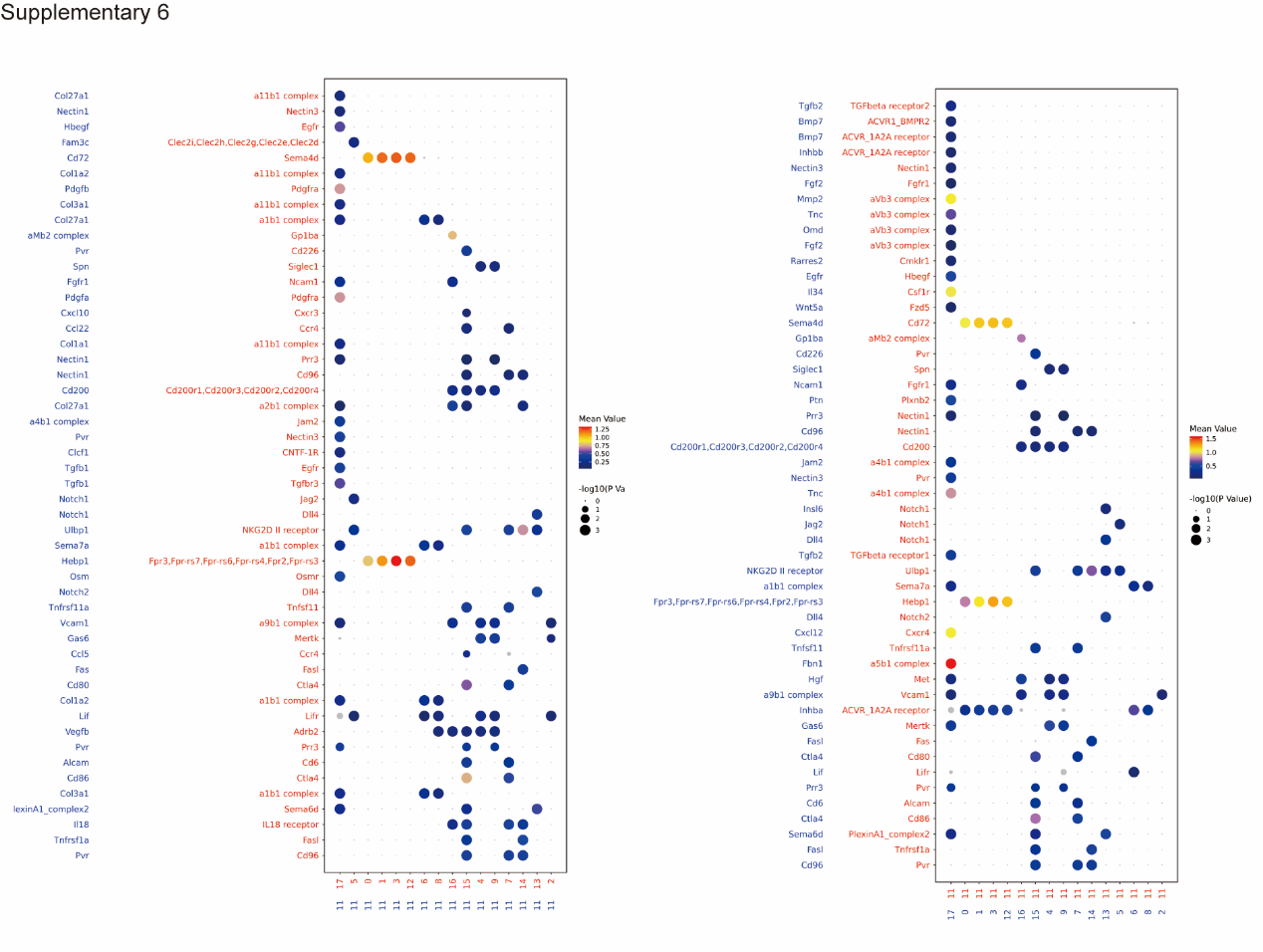


Supplementary 7: Dot plot showed selected ligand-receptor interactions between cluster2 and immune cells.


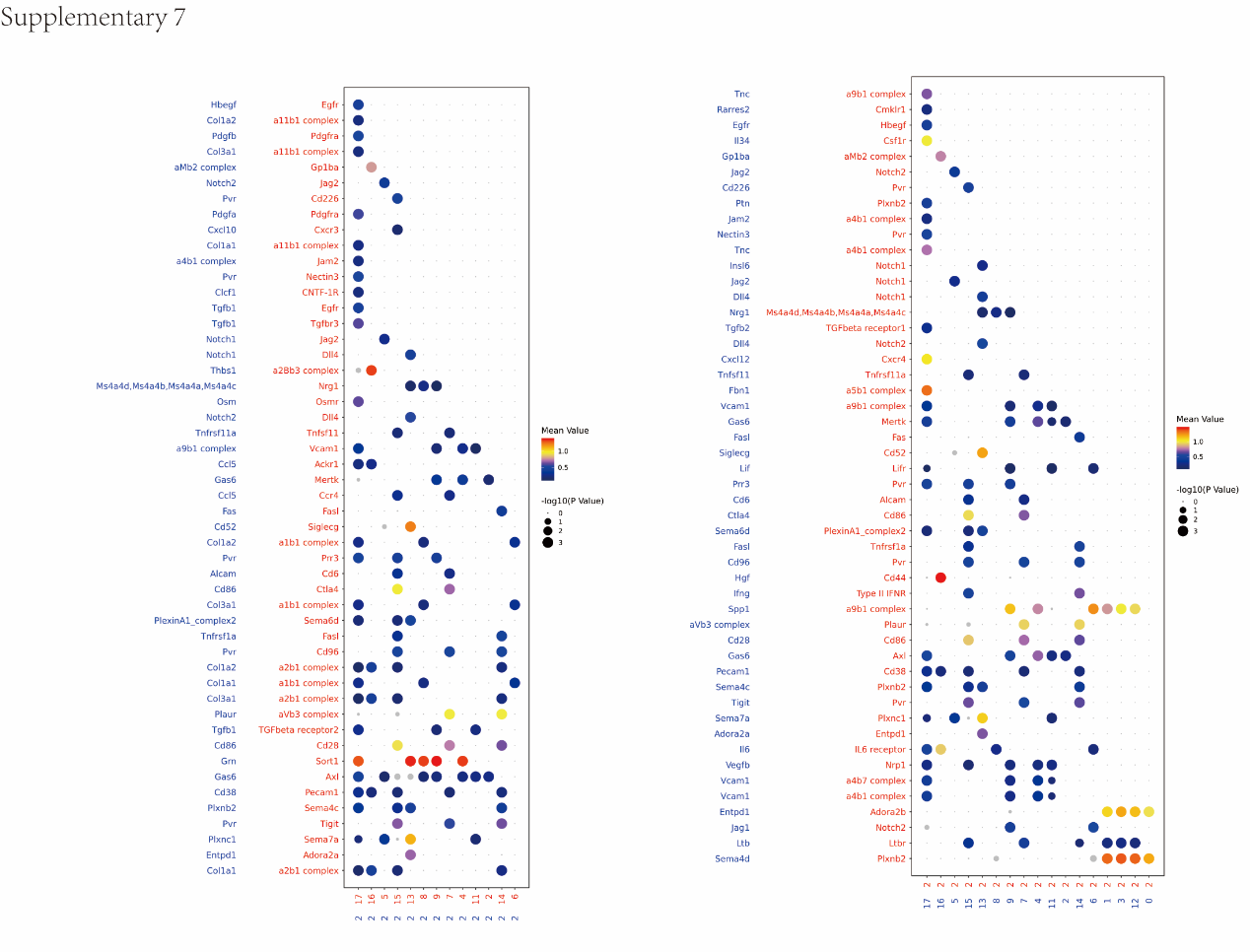


Supplementary 8: Violin plot view top 20 marker gene demonstrating overall gene expression of cluster17.


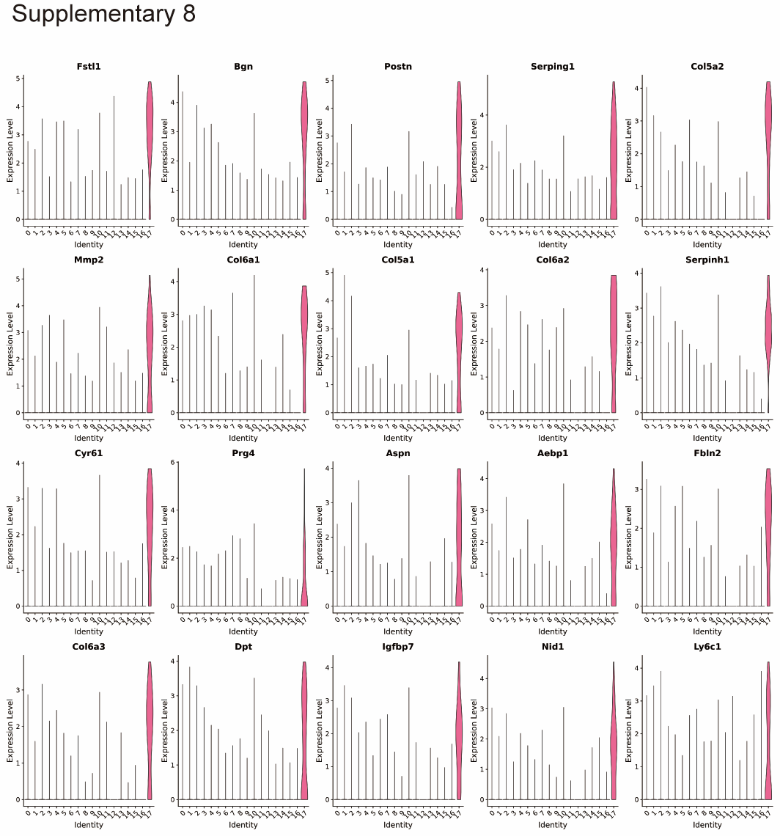


Supplementary 9: GO analysis of the differential genes in cluster 2 at day 3 in two wound groups and KEGG analysis of downregulated gene enrichment at day 5 in the diabetes group.


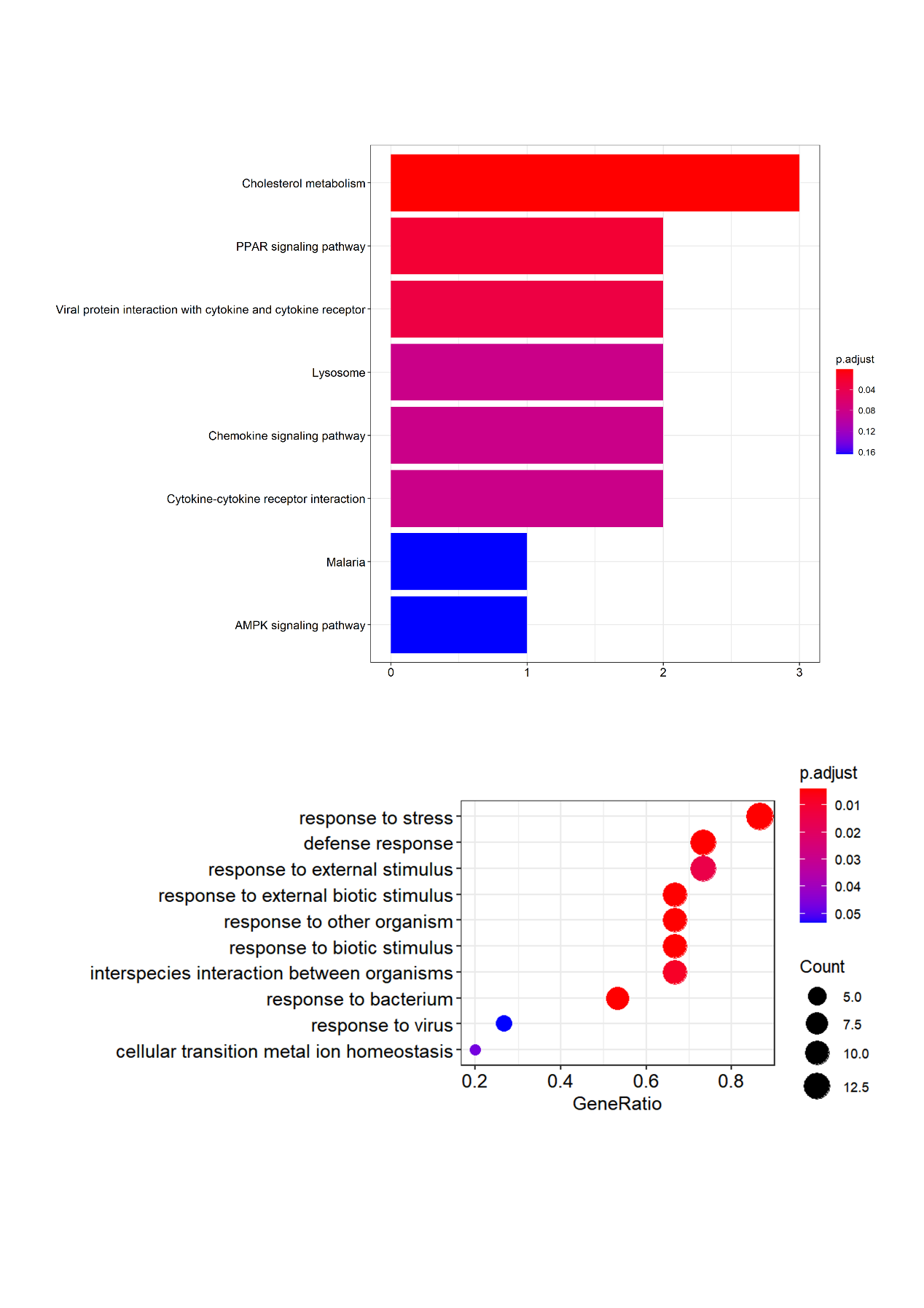


Supplementary 10: GO enrichment analysis of the differential genes in cluster 4 at day 3 and the biological functions of the differences.


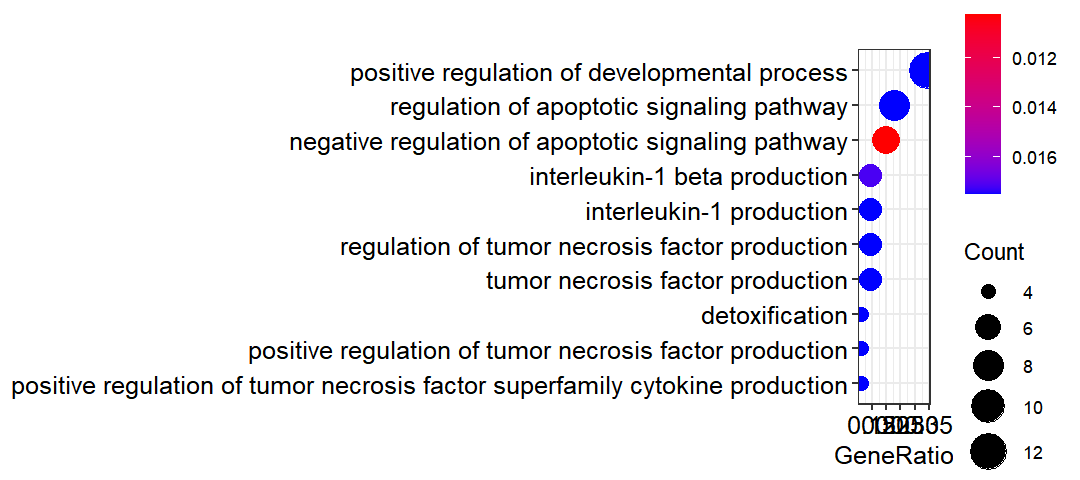


Supplementary 11: GO and KEGG analysis of cluster 9 cells in different time points.


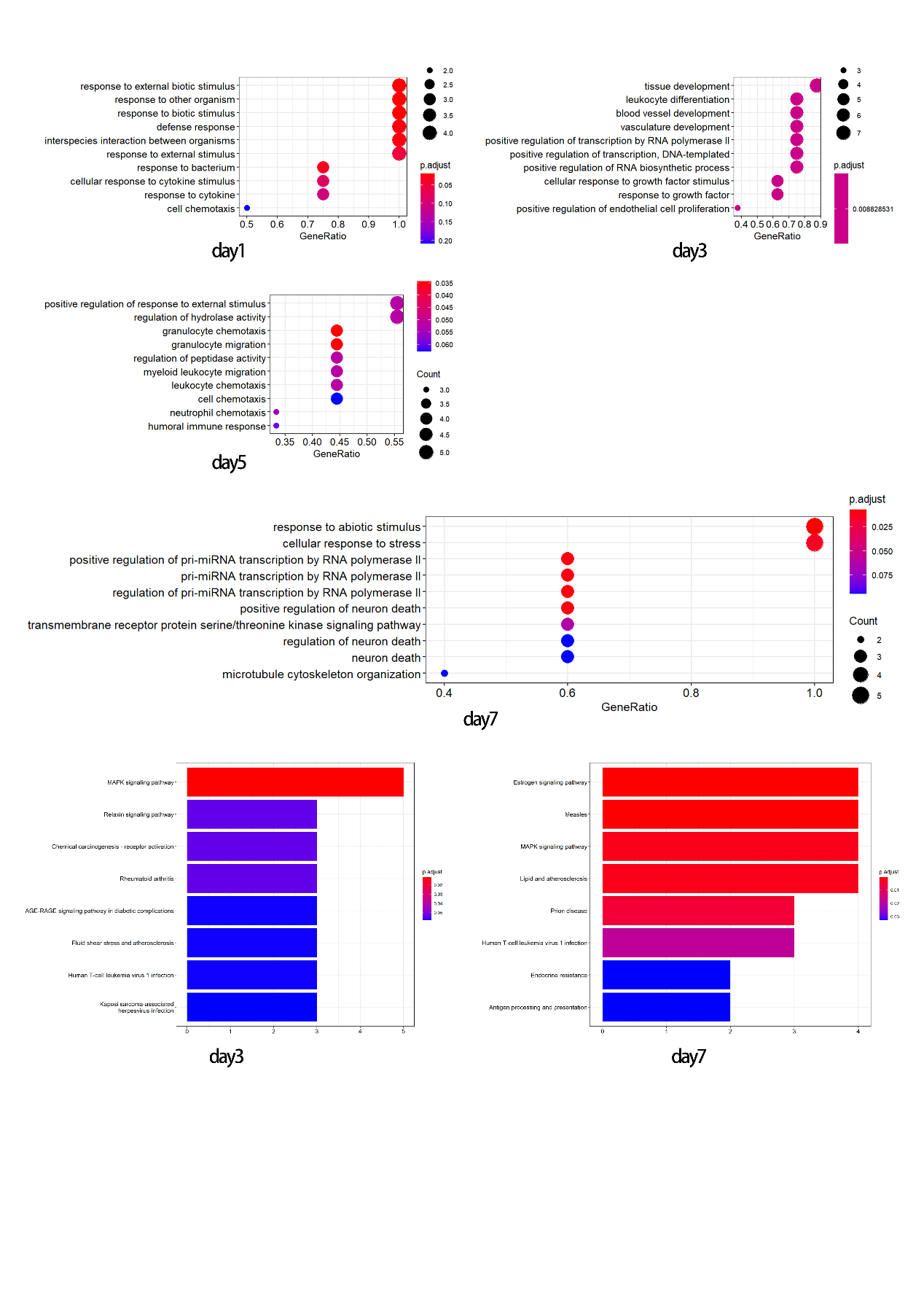

Supplement: Supplementary file 1 — Supplementary Material 1 [file 12079_2022_707_MOESM1_ESM.docx]
